# Directed evolution of drug-like Aβ conformation-specific antibodies

**DOI:** 10.3389/fimmu.2025.1655893

**Published:** 2025-10-13

**Authors:** Alec A. Desai, Matthew D. Smith, Jennifer M. Zupancic, Emily K. Makowski, Yulei Zhang, Julia E. Gerson, Shannon J. Moore, Alexandra B. Sutter, Sean P. Ferris, Magdalena I. Ivanova, Geoffrey G. Murphy, Henry L. Paulson, Peter M. Tessier

**Affiliations:** ^1^ Department of Chemical Engineering, University of Michigan, Ann Arbor, MI, United States; ^2^ Biointerfaces Institute, University of Michigan, Ann Arbor, MI, United States; ^3^ Department of Pharmaceutical Sciences, University of Michigan, Ann Arbor, MI, United States; ^4^ Department of Neurology, University of Michigan, Ann Arbor, MI, United States; ^5^ Protein Folding Disease Initiative, University of Michigan, Ann Arbor, MI, United States; ^6^ Department of Molecular and Integrative Physiology, University of Michigan, Ann Arbor, MI, United States; ^7^ Department of Pathology, University of Michigan, Ann Arbor, MI, United States; ^8^ Biophysics Program, University of Michigan, Ann Arbor, MI, United States; ^9^ Michigan Neuroscience Institute, University of Michigan, Ann Arbor, MI, United States; ^10^ Michigan Alzheimer’s Disease Center, University of Michigan, Ann Arbor, MI, United States; ^11^ Department of Biomedical Engineering, University of Michigan, Ann Arbor, MI, United States

**Keywords:** amyloid, conformational, fibril, aggregation, antibody, mAb, affinity maturation, Alzheimer’s disease

## Abstract

Monoclonal antibodies that recognize conformational epitopes in protein aggregates are important for research, diagnostic, and therapeutic applications related to neurodegenerative disorders such as Alzheimer’s and Parkinson’s diseases. Unfortunately, it remains challenging to discover and engineer high-quality conformational antibodies that are specific for protein aggregates and possess optimal combinations of three key binding properties, namely high affinity, high conformational specificity, and low off-target binding. Here we report a directed evolution approach for generating high-quality conformational antibodies against Alzheimer’s Aβ fibrils in the native IgG format. Our directed evolution approach uses targeted mutagenesis, yeast surface display, cell sorting, and deep sequencing to identify antibody candidates with optimized binding properties. Notably, we find that this approach yields robust isolation of IgGs with higher affinity, higher conformational specificity, and lower off-target binding than multiple clinical-stage Aβ antibodies, including aducanumab and crenezumab. This antibody engineering platform can be readily applied to generate conformational antibodies against diverse types of peptide and protein aggregates linked to human diseases.

## 1 Introduction

Many neurodegenerative diseases are strongly linked to protein misfolding and assembly into diverse types of protein aggregates, ranging from small oligomers to large amyloid fibrils (1). The diversity of protein aggregates that can form from a single protein is vast and requires molecular agents with extreme conformational specificity for detection, diagnostic, and therapeutic applications. Monoclonal antibodies are the leading agents for such applications, given their high affinity and specificity for diverse types of protein antigens.

In particular, three general types of antibodies have been reported for specific recognition of amyloid-forming proteins. The first antibody type possesses both sequence and conformational specificity (2–8). These antibodies are sequence-specific because they recognize only one target amyloid-forming protein, and they are conformation-specific because they recognize a specific 3D conformation of their cognate protein that is absent in the monomeric protein. The second antibody type possesses conformational specificity and lacks sequence specificity (9–12). These antibodies recognize a common conformational motif in protein aggregates formed by different amyloid-forming proteins. The third antibody type possesses sequence specificity but lacks conformational specificity, resulting in antibody recognition of both monomeric and aggregated forms of the target amyloid-forming proteins (13–15). These antibodies are commonly referred to as “pan” or “total” antibodies.

The first antibody type with both sequence and conformational specificity is generally the most valuable, which has motivated the generation of such antibodies against diverse amyloid-forming proteins (4, 6, 7). Most previous approaches either use immunization followed by hybridoma, phage, or single B-cell screening, or *in vitro* nonimmune libraries and phage or yeast surface display screening. These approaches have been widely used to generate diverse antibodies with combinations of desirable properties. Nevertheless, they also have several disadvantages, including the generation of non-conformational antibodies in addition to conformational ones and the need for extensive secondary screening to identify rare variants with desirable combinations of high affinity, conformational specificity, and sequence specificity, as well as low off-target binding.

**Figure 1:**
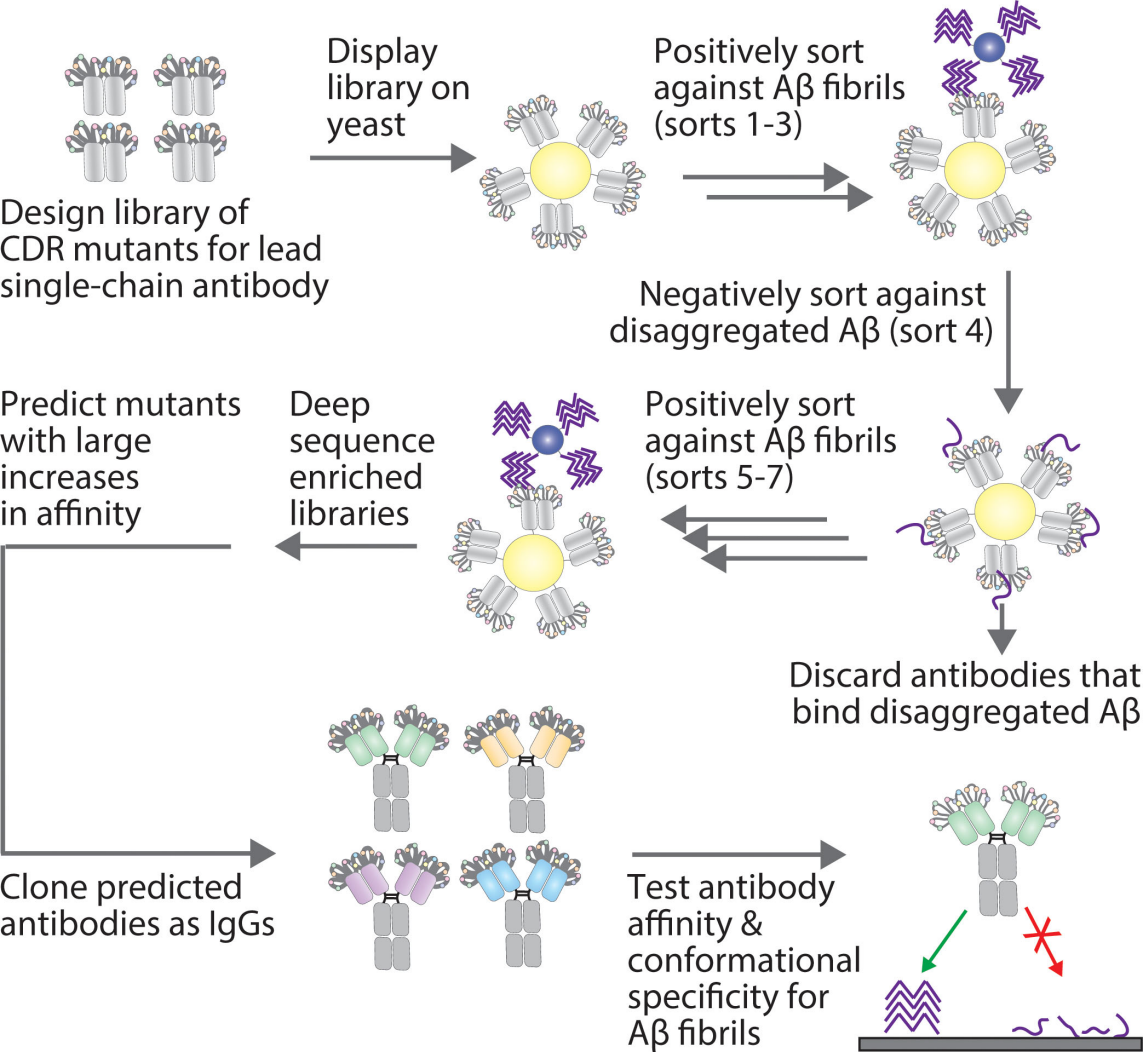
Overview of the approach for affinity maturing Aβ conformational antibodies. A lead Aβ conformational antibody, in a single-chain variable fragment (scFv) format, was affinity matured by first generating a sub-library via site-specific (degenerate codon) mutagenesis at ten sites in two CDRs (light chain CDR2 and heavy chain CDR1). Next, the antibody library was displayed on the surface of yeast and sorted positively for binding to Aβ fibrils via magnetic-activated cell sorting and negatively for a lack of binding to disaggregated Aβ via fluorescence-activated cell sorting. The resulting enriched libraries were deep sequenced, and multiple methods were tested for predicting antibody mutants with large increases in affinity. The predicted antibodies were then cloned as IgGs and tested for their affinity and conformational specificity for Aβ aggregates using synthetic and biological samples.

Here we report a simple and predictable approach for increasing the affinity of a lead antibody specific for fibrils of the Alzheimer’s Aβ peptide (**Figure 1**). This approach incorporates the design of multisite mutation antibody libraries, *in vitro* library screening using yeast surface display, deep sequencing and a straightforward scoring method to identify antibody variants with increased affinity. This results in high-confidence predictions of antibody variants with increased affinity, eliminates the need for secondary screening to identify optimized variants, and yields antibodies with superior binding properties relative to multiple clinical-stage Aβ antibodies.

## 2 Materials and methods

### 2.1 Antibody library generation

A previously reported Aβ conformational antibody (clone 97) (3) was cloned in a yeast surface display plasmid (pCTCON2 with modified linker with Aga2 and antibody gene) in the single-chain variable fragment (scFv) format, in the V_L_-V_H_ orientation, as a C-terminal fusion to Aga2 (Aga2-scFv). Next, ten sites in the CDRs (complementarity determining regions) of the antibody were diversified using NNK mutagenesis, which included diversifying five sites in heavy chain CDR1 (HCDR1; H27, H31, H32, H33, and H34) and five sites in light chain CDR2 (LCDR2; L50, L51, L52, L53, and L55). The library generation and transformation were performed as described earlier (3, 6), and ~5x10^8^ transformants were obtained.

Sites for mutagenesis were chosen by evaluating the diversity of each residue of the CDRs in human antibody repertoires that were not previously mutated, HCDR1 and LCDR2. Residues in these CDRs were chosen for mutagenesis if the position had tyrosine or aspartic acid frequencies of >2% in the abYsis database for human antibodies (16). Sites that met these criteria were then ranked in order of decreasing wild-type frequency. This led to the identification of ten sites, with the maximum observed WT frequency being 65%.

### 2.2 Antigen preparation

Aβ42 peptide was obtained from Anaspec (AS-20276), and Aβ fibrils were assembled as described previously (3, 6). Briefly, 1% biotinylated Aβ monomer (Anaspec, AS-23524-01) was incubated with non-biotinylated Aβ monomer for 3–5 d and purified via ultracentrifugation at 221,000 xg at 4 °C for 1 h. The fibril pellet was then resuspended in an equivalent volume of PBS and frozen at -80 °C until further use. For magnetic-activated cell sorting (MACS), Aβ fibril-coated beads were prepared by first sonicating fibrils on ice for 2 min (10 s on, 30 s off), and then incubating with streptavidin-coated Dynabeads (Invitrogen, 11047) at a final concentration of 1 µM (final volume of 400 µL for 10^7^ beads) at room temperature for 2–3 d with end-over-end mixing. For bead-based affinity analysis, the beads were prepared in a manner similar to that described above but were incubated with 0.3 or 1 nM Aβ fibrils.

### 2.3 Antibody library sorting

Yeast cells (~10^9^) displaying antibodies were washed twice with PBS with 1 g/L BSA (PBSB) by centrifuging at 2500 xg for 5 min. Streptavidin Dynabeads (~10^7^) coated with immobilized Aβ fibrils were washed twice with PBSB in 1.5 mL tubes after placing the columns on a magnetic stand. The beads and yeast cells were incubated in a final volume of 5 mL in PBSB with 1% milk at room temperature for 3 h with end-over-end mixing. Post incubation, the beads were washed once with ice-cold PBSB by placing the 1.5 mL tube on a magnet, which was followed by recovering bound yeast in 50 mL of SDCAA media (16.75 g/L sodium citrate trihydrate, 4 g/L citric acid, 5 g/L casamino acid, 3.7 g/L yeast nitrogen base without amino acids and 20 g/L glucose) at 30 °C for two days with agitation. Dilutions were plated on yeast dropout plates to determine the number of yeast cells retained during the selection.

In rounds 2 and 3, 10^7^ yeast cells displaying antibodies were washed twice with PBSB. In parallel, ~10^7^ streptavidin beads were washed twice with PBSB in 1.5 mL by placing them on a magnet. Beads and cells were incubated in a final volume of 1 mL in 1% milk in PBSB at room temperature for 3 h with end-over-end mixing. Post incubation, the tube was placed on a magnet and unbound yeast cells were removed. The beads were washed once with ice-cold PBSB by placing it on the magnet again. Yeast bound to beads were recovered and grown in 50 mL of SDCAA media, as described above. Sort 4 was a negative selection against biotinylated disaggregated Aβ peptide. Yeast cells (10^7^) were washed twice with PBSB and incubated with disaggregated Aβ42 peptide (1 μM) at room temperature for 3 h with end-over-end mixing in the presence of a 1000x dilution of mouse anti-myc antibody (Cell Signaling; 2276S). Post-primary incubation, the cells were washed once with ice-cold PBSB and then incubated with 200x dilution of goat anti-mouse IgG AF488 (Invitrogen, A11001) and 1000x dilution of streptavidin AF647 (Invitrogen, S32357) on ice for 4 min. Post-secondary incubation, the cells were washed once with ice-cold PBSB and sorted on a Beckman Coulter MoFlo Astrios sorter. Yeast cells displaying antibodies, but showing minimal binding to disaggregated Aβ, were collected and grown in SDCAA media. Sort 5, which was a positive MACS sort against Aβ fibrils, was performed similarly to sorts 2 and 3. Sorts 6–8 were also performed similarly to sorts 2–3 except that the yeast bound to fibril-coated beads (post incubation) were washed thoroughly thrice with PBSB-T (PBSB with 0.05% v/v Tween-20) via end-over-end mixing for 10–20 min (10 min for sort 6 and 20 min for sort 7 and 8) per wash (room temperature) in an attempt to select for antibodies with higher affinities and slow off-rates.

### 2.4 Antibody library deep sequencing

Plasmids were recovered from five rounds of sorting using a yeast mini prep kit (Zymo, D2004). The five rounds selected for deep sequencing were rounds two, three, five, six, and seven. All five of these sorts were positive selections against Aβ fibrils using MACS. The deep sequencing sample preparation was completed in a two-step PCR. First, light chain framework 2 through heavy chain framework 2 was amplified from the plasmids with primers that added the necessary adapter sequences for Illumina sequencing. The primers also incorporated a single base pair shift in each sample to decrease sequence homology during sequencing (17). The PCR product was purified from a 1% agarose gel using a QIAquick Gel Extraction Kit (Qiagen, 28704). The second PCR used 2 µL of this purified product with primers identical to the Illumina adapter regions. This product was also run on a 1% agarose gel and purified as before. The concentration of each sample was then determined using a Qubit 4 Fluorometer using the 1x dsDNA High Sensitivity Assay Kit (Thermo Fisher Scientific, Q33230). The samples were mixed in equimolar proportions and submitted for a 300 bp paired-end MiSeq run. The sequencing was performed in duplicate, and each set of samples was sequenced independently.

The resulting sequence files were analyzed by first merging the two fastq files for each sample into a single file using BBMerge (18). The single fastq file was then converted to a fasta file for convenience. This file was then analyzed for correct sequence reads by identifying sequences that were the correct length and started with the correct six amino acids. Sequences with one amino acid change in the first 6 amino acids were also considered correct. The amino acids of the 10 mutated positions were then extracted from the full sequence and recorded as the mutation string. The number of occurrences of each unique mutation strings was recorded. The code for analyzing the sequencing data is available upon request.

### 2.5 Bioinformatics analysis of deep sequencing data

Sequences that were common to both replicates for any round (two, three, five, six, or seven) were collected and the frequency of each clone was averaged across the two replicates. For rounds in which a clone was not observed, its frequency was set to zero. This resulted in >8000 unique sequences. Each sequence therefore had five frequency observations corresponding to its abundance in rounds two, three, five, six, and seven. To select sequences for further evaluation, position-specific scoring matrices (PSSMs) were calculated for sequences from rounds five, six, and seven. First, a count matrix of each amino acid in each position was created using the count of each sequence in the input data matrix. For example, the sequence SASFYYATYI, which had 8 observations in round 5, contributed 8 counts of “S” in position 1, “A” in position 2, and so on in the round five count matrix. Second, the count matrix was converted to a position probability matrix (PPM) by adding pseudocounts to each amino acid at every position and normalizing by the total number of counts and pseudocounts. The pseudocount was set to the square root of the total number of sequences observed in the round of interest (19, 20).

Next, the PSSM was created by taking the log2 of each element of the PPM divided by the background probability, which was set at 5%. Finally, PSERMs were computed for round seven (the PSSM of round seven minus the PSSM of round six) and round six (the PSSM of round six minus the PSSM of round five). The set of >8000 clones was then restricted to sequences common to both replicates in rounds two, three, five, and six, allowing sequences to be unobserved in round seven, with their frequency set to zero. This resulted in 244 unique sequences. Each antibody sequence was then scored using the two PSERMs, as described previously (21). The sequences that were in the top 40 clones for both PSERM matrices were considered for further analysis. The overlap resulted in 23 clones, of which 7 were removed because they contained an unpaired cysteine.

To compare the PSERM scores to more conventional metrics, the mAb affinities were compared to the frequency of each clone in the final round of sorting (R7), the global enrichment ratio ( $\log_{2}\left(f_{i,R7}/f_{i,R2}\right)$ ), and the local enrichment ratio ( $\log_{2}\left(f_{i,R7}/f_{i,R6}\right)$ ). The top 40 scoring clones for each metric (excluding WT) are reported in **Supplementary Figure S6**.

### 2.6 Antibody cloning and production

Variable domains of antibodies selected from deep sequencing analysis were ordered as geneblocks (Integrated DNA Technologies). The geneblocks were amplified by PCR using forward and reverse primers containing EcoRI and NheI (variable heavy, V_H_) or BsiWI (variable light, V_L_) restriction sites. The PCR products were purified via agarose gel electrophoresis and then digested with *EcoRI-HF* (New England Biolabs, R3101L) and *NheI-HF* (New England Biolabs, R2121L) for V_H_ or *BsiWI-HF* (New England Biolabs, R3553L) for V_L_ as per manufacturer’s protocol and finally purified using a PCR clean up kit (Qiagen, 28104).

To facilitate cloning of the digested antibody genes into antibody heavy and light chain mammalian expression plasmids (pTT5) with human IgG1 and kappa framework, the expression plasmids were first digested with *EcoRI-HF* and *NheI-HF* (heavy chain plasmid) or *BsiWI-HF* (light chain plasmid). Next, the digested plasmids were treated with calf intestinal alkaline phosphatase (New England Biolabs, M0525L) and purified via agarose gel electrophoresis. Finally, the antibody genes were ligated into the linearized backbone of the expression plasmids with T4 DNA ligase (New England Biolabs, M0202L), transformed into competent DH5α cells, plated on LB-agar plates (supplemented with 100 µg/mL ampicillin), and grown overnight at 37 °C. The next day, individual colonies were picked, grown in 5 mL of LB media (supplemented with 100 µg/mL ampicillin) overnight at 37 °C, mini prepped (Qiagen, 27106), and sequence confirmed by Sanger sequencing.

Antibodies were expressed in suspension HEK293-6E cells (National Research Council Canada). Cells were maintained and passaged in F17 media (Gibco, A1383501) and supplemented with 30 mg/L glutamine (Invitrogen, A1383502), 10% kolliphor (Fisher, NC0917244), and geneticin (Gibco, 10131035). Cells were transfected with ~15 µg of plasmid (7.5 μg of heavy chain and 7.5 μg of light chain) and ~45 µg of PEI at a density of 1.7–2 million cells/mL. Yeastolate (BD Sciences, 292804) was added at 20% w/v 24–48 h post-transfection, and cells were allowed to grow for another 3–5 d. Post expression, cells were centrifuged at 3500 xg for 40 min, media/supernatant was collected and transferred to a new tube, and incubated with 0.5 mL Protein A agarose resin (Pierce, 20333) overnight at 4 °C with gentle agitation. Protein A resin was collected in a filter column (Fisher, 89898) under vacuum and washed with 50 mL of PBS. Proteins were eluted from resin with 0.1 M glycine (pH 3.0), buffer exchanged into 20 mM acetate (pH 5.0) using Zeba desalting columns (Fisher, 89894), and filtered through 0.22 µm syringe filters (Fisher, SLGV004SL). Protein concentrations were evaluated by measuring absorbance at 280 nm, and purity was evaluated by SDS-PAGE (Invitrogen, WG1203BOX) and analytical size-exclusion chromatography.

### 2.7 Antibody affinity and conformational specificity analysis

Affinity analysis for antibody binding to Aβ42 fibrils was performed using a bead-based assay in which 1% biotinylated Aβ fibrils were immobilized on streptavidin Dynabeads at 0.3 μM. Beads were blocked with 10% milk in PBSB at room temperature for 1 h with end-over-end mixing and washed once with PBSB. Antibodies to be tested were either used fresh or were thawed and centrifuged at 21,000 xg for 5 min in a tabletop centrifuge. The supernatant of the antibody solution was transferred to a fresh tube, and the antibody concentration was evaluated by measuring the absorbance at 280 nm. Next, after the beads were washed, they were incubated with antibodies at varying concentrations in PBSB with 1% milk for 3 h at room temperature with mild agitation. Post-primary incubation, beads were washed once with ice-cold PBSB and incubated with 300x dilution of goat anti-human Fc AF647 (Jackson ImmunoResearch, 109-605-098) on ice for 4 min. Following secondary incubation, beads were washed once with ice-cold PBSB and analyzed on Bio-Rad Ze5 flow cytometer. As a control, blank streptavidin beads (without immobilized Aβ fibrils) were processed in the same manner.

Conformational specificity for antibody binding to Aβ fibrils in the presence of disaggregated Aβ was also evaluated with a bead-based assay. First, the antibodies (10 nM) were preincubated with disaggregated Aβ (0.1–1000 nM) for 5–10 min at room temperature. Next, the antibody/Aβ mixture was incubated with streptavidin Dynabeads coated with immobilized Aβ fibrils (1 μM, 1% biotinylated peptide) in PBSB with 1% milk at room temperature for 3 h with mild agitation. Finally, the beads were washed, incubated with secondary detection reagents, and the relative amount of bound antibody was evaluated using flow cytometry in a manner similar to that described for the antibody affinity analysis.

### 2.8 Transgenic mouse model and brain tissue processing

This study was performed in a facility approved by the American Association for the Accreditation of Laboratory Animal Care, and the experiments were conducted in accordance with the NIH Guide for the Care and Use of Laboratory Animals and approved by the Institutional Animal Care and Use Committee of the University of Michigan, as described previously (3). Briefly, mice were housed and maintained according to U.S. Department of Agriculture standards (12 h light/dark cycle with food and water available ad libitum). 5xFAD mice (B6.Cg_Tg(APPSwFlLon,PSEN1*M146L*L286V)6799Vas/Mmjax; The Jackson Laboratory MMRRC stock #034848) expressing human amyloid precursor protein (APP) and presenilin-1 (PSEN1) with five AD mutations [the Swedish (K670N/M671L), Florida (I716V), and London (V717I) APP mutations and the M146L and L286V PSEN1 mutations] and non-transgenic littermates (courtesy of Geoffrey Murphy, University of Michigan) were euthanized at 8 months (for immunofluorescence analysis) and 22–24 months (for immunoblots and western blots) for brain collection.

Animals were deeply anesthetized with isoflurane and perfused transcardially with 1x PBS, as described previously (3). Briefly, brains were divided sagittally, with one half frozen at -80 °C for biochemical studies while the other half was fixed (4% paraformaldehyde) and cryoprotected. Fixed hemispheres were snap frozen and sectioned at 12 μm sagittally using a cryostat.

### 2.9 Immunoblotting, western blotting, and immunofluorescence analysis of mouse brain samples

5xFAD and non-transgenic littermate control forebrain samples were homogenized in PBS, and pellets were resuspended in radioimmunoprecipitation assay (RIPA) buffer with protease inhibitor, as described previously (3). RIPA (insoluble) fractions of brain extracts (7 µg of total protein) were spotted directly onto nitrocellulose membranes and allowed to dry (1 h). Dot blots were blocked with 10% nonfat dry milk in Tris Buffered Saline with 0.1% Tween 20 (TBST) buffer at room temperature (1 h). Each dot blot was then incubated with antibodies at 50 nM (1% nonfat dry milk in TBST) overnight at 4 °C. Next, the blots were washed with TBST and incubated with a 5000x diluted solution of HRP-conjugated goat anti-human IgG at room temperature for 1 h. Afterward, the blots were washed with TBST, developed using Ecobright Nano HRP Substrate (Innovative Solutions), and visualized with the Genesys G:Box imaging system (Syngene). Control dot blots (loading controls) were stained with Ponceau S (5 min) and washed 3x with distilled water. Three independent repeats were performed.

For western blotting, 50 µg of total protein was loaded on precast NuPAGE 4-12% Bis-Tris gels (Invitrogen, WG1403BOX). Gels were subsequently transferred onto nitrocellulose membranes and first stained with Ponceau S and washed 3x with distilled water. After imaging, membranes were destained for 1 min with 0.1 M NaOH and washed 3x with distilled water. Next, membranes were blocked for 1 h at room temperature with 10% nonfat dry milk in TBST buffer. Membranes were probed overnight at 4°C with clones in 1% nonfat dry milk in TBST. HRP-conjugated goat anti-human IgG (5000x dilution) was used for detection. Ecobright Nano HRP Substrate (Innovative Solutions, EBNH100) was used to visualize bands with the Genesys G:Box imaging system (Syngene). Three independent repeats were performed.

Fixed brain sections were post-fixed for 10 min in methanol at 4 °C. Sections were washed in 1x PBS three times for 10 min, and heat-induced antigen retrieval in 10 mM Citrate Buffer (pH 6) was performed by microwave. Sections were washed in 1x PBS two times for 5 min and permeabilized with 0.5% Triton-X 100, washed for 10 min in 1x PBS, and blocked using the Mouse on Mouse (M.O.M.) Mouse IgG Blocking Reagent (M.O.M. Immunodetection Kit, Vector, BMK-2202) for 1 h. Sections were washed twice for 2 min in 1x PBS and incubated for 5 min in M.O.M. diluent. Sections were then incubated with the engineered antibodies in this study (10 nM) and anti-Aβ NAB228 (1:200) in M.O.M. diluent overnight at 4°C. The following day, sections were washed in 1x PBS three times for 10 min each and incubated with goat anti-mouse IgG Alexa-488 (Invitrogen; 1:500) and anti-human IgG Alexa-647 (1:500) for 1 h. Sections were then washed in PBS three times for 10 min each and incubated with DAPI (Sigma) to label nuclei for 5 min at room temperature, washed three times for 5 min each, and mounted with Prolong Gold Antifade Reagent (Invitrogen). Slides were imaged using a Leica SP5 Confocal microscope.

### 2.10 Human disease brain tissue

Frozen brain tissues from the hippocampus of subjects with Alzheimer’s disease and age-matched controls were obtained from the Michigan Brain Bank (University of Michigan, Ann Arbor, MI, USA) and prepared as described previously (3). Protocols were approved by the Institutional Review Board of the University of Michigan and abide by the Declaration of Helsinki principles. Brain tissue was collected with the informed consent of patients.

### 2.11 Immunoblotting analysis of human brain samples

The human brain tissue was homogenized and processed as described previously (3). Next, the total protein concentration for each processed sample was measured using BCA (Fisher, 23225). Samples were then diluted to 1 µg/µL (total protein concentration) and deposited (1 µL) on nitrocellulose membranes (at several dilutions) and allowed to dry at room temperature for at least 2 h. The conformational antibodies were first cloned with mouse IgG2a Fc in the same mammalian expression plasmid. The resulting antibodies were chimeric, comprising human Fab constant domains (C_L_ and C_H_1) and mouse IgG2a Fc (C_H_2, C_H_3). Membranes were blocked with 10% milk in PBS for 1 h at room temperature and incubated with mAbs developed in this work (50 nM), aducanumab (5 nM) and NAB228 (1000x dilution) in 1% milk in PBST (PBS with 0.1% v/v Tween-20) overnight at 4 °C. The next day, the blots were washed 3x with PBST and incubated with 10,000x dilution of goat anti-mouse IgG HRP (Invitrogen, 62-6520) at room temperature for 1 h. Following secondary incubation, the blots were washed 3x with PBST. The blots were then incubated with ECL (Fisher, 32109), and signals were captured on X-Ray film (Fisher, 34090).

### 2.12 Immunohistochemistry analysis of human brain samples

Tissue from the frontal cortex of patients with high Alzheimer’s disease neuropathologic change and moderate cerebral amyloid angiopathy was obtained as paraffin-embedded blocks from the Michigan Brain Bank. The University of Michigan Rogel Cancer Center Tissue and Molecular Pathology Shared Resource (TMPSR) Core performed all immunohistochemical staining using a DAKO Autostainer Link 48 (Agilent, Carpiteria, CA). Aducanumab and 97A34 were tagged with Digoxigenin, and detection was performed using a Human-on-Human HRP-Polymer kit (Biocore Medical, BRR4056KG). Detection of NAB228 was done with an anti-mouse/rabbit Flex HRP kit (Agilent).

Staining of the brain sections was done as previously described (22). Briefly, the paraffin was removed from the blocks with xylene, and then the brain sections were rehydrated and rinsed in TBST. Heat-induced epitope retrieval was performed for aducanumab and 97A34 using Dako Envision Flex TRS (low pH), followed by blocking with peroxidase block for 5 min. Aducanumab and 97A34 were applied to their slides for 1 h. NAB228 was applied for 30 min. Secondary antibody was then applied and incubated for 15 min for aducanumab and 97A34 (mouse anti-Digoxigen) and for 20 min for NAB228 (goat anti-mouse/rabbit flex HRP). After washing with TBST, MACH2 mouse HRP polymer was applied to the aducanumab and 97A34 slides for 30 min. Following a rinse with TBST, 3,3’diaminobenzidine (DAB) was added to all slides for 10 min. After a rinse with DI water, the slides were counterstained for nuclei detection with hematoxylin. The final processing of the slides was performed as described above.

### 2.13 Polyspecificity analysis

The polyspecificity reagent (SMP) was prepared as previously described (23, 24). CHO cells (10^9^, Gibco, A29133) were pelleted, the cell pellets were washed separately with PBSB and Buffer B (50 mM HEPES, 0.15 M NaCl, 2 mM CaCl_2_, 5 mM KCl, 5 mM MgCl_2_, 10% Glycerol, pH 7.2), and then pelleted again. The pellets were resuspended in Buffer B supplemented with a protease inhibitor (Sigma Aldrich, 4693159001). The resuspended cells were homogenized for 90 s (three cycles of 30 s), spun down at 40,000 xg for 1 h, and the supernatant was discarded.

The pellet, which contained the membrane proteins, was resuspended in Buffer B with a Dounce homogenizer. The total protein concentration was evaluated using a detergent-compatible protein assay kit (BioRad, 5000116). The suspension was diluted to a theoretical concentration of 1 mg/mL in solubilization buffer (pH 7.2), containing 50 mM HEPES, 0.15 M NaCl, 2 mM CaCl_2_, 5 mM KCl, 5 mM MgCl_2_, 1% n-dodecyl-β-D-maltopyranoside (Sigma Aldrich, D4641), and a protease inhibitor (Sigma Aldrich, 11873580001). The solution was then mixed overnight at 4 °C, rotating end-over-end. The soluble membrane protein (SMP) containing fraction was centrifuged at 40000 xg for 1 h, and the supernatant was collected. The final concentration of the supernatant was diluted to 1.0 mg/mL.

Sulfo-NHS-LC-biotin (Thermo Fisher, PI21335) was dissolved in distilled water at ~11.5 mg/mL. Stock solution of Sulfo-NHS-LC-biotin (150 µL) and the SMP reagent (4.5 mL at 0.8-0.9 mg/mL) were mixed via end-over-end mixing at room temperature (45 min). The reaction was quenched (10 µL of 1.5 M hydroxylamine at pH 7.2), and biotinylated SMP was aliquoted at 1 mg/mL (total protein concentration) and stored at -80 °C.

The polyspecificity assay was performed as previously described (3, 23). Protein A magnetic beads (Invitrogen, 88846) were washed with PBSB and incubated with antibodies overnight at 4 °C. Next, the protein-coated beads were washed and resuspended with a 10x diluted solution of biotinylated SMP (~0.1 mg/mL) and incubated on ice for 20 min. Beads were again washed with PBSB and incubated with streptavidin AF-647 (Invitrogen, S32357) and 1000x diluted solution of goat anti-human Fc F(ab’)_2_ AF-488 (Invitrogen, H10120) on ice (4 min). Beads were washed, resuspended in PBSB, and analyzed via flow cytometry. Three independent repeats were performed with all results normalized between a low specificity control, emibetuzuab, and a high specificity control, elotuzumab.

### 2.14 Analytical size-exclusion chromatography

Antibodies in this study were evaluated via analytical size-exclusion chromatography (SEC) with a Shimadzu Prominence HPLC System outfitted with a LC-20AT pump and SIL-20AC autosampler. After purification with Protein A, antibodies were buffer exchanged into PBS and 100 μL of 0.1 mg/mL protein were loaded onto a SEC column (Superdex 200 Increase 10/300 GL column; GE, 28990944) and analyzed at 0.75 mL/min using a PBS running buffer supplemented with 200 mM arginine (pH 7.4). Absorbance was monitored at 220 and 280 nm, and the 280 nm signal was primarily used for analysis. The percentage of antibody monomer was calculated by analyzing absorbance peaks between the void and column elution times (~8–22 min).

### 2.15 Antibody melting temperature analysis

Differential scanning fluorimetry was performed to determine the antibody melting temperatures. mAbs were first diluted to a concentration of 0.12 mg/mL in 1x PBS. Diluted mAbs were then mixed at a 7:1 volume ratio with diluted Protein Thermal Shift Dye (Applied Biosystems, 4461146) to achieve a final concentration of 1x dye. 1x PBS was mixed with dye at the same ratio to measure the background signal. mAb-dye and PBS-dye solutions were added to individual wells of a clear 384-well plate. Samples were then submitted to the University of Michigan Advanced Genomics core for analysis. Each plate was centrifuged at 1000–2000 rpm for 1 min prior to analysis. Plates were then inserted into an ABI Prism 7900HT Sequence Detection System (Applied Biosystems). The fluorescence signals from the sample and background wells were then measured between 25 and 98 °C. For data analysis, background signal was subtracted from the sample signal at each temperature. The background signal was determined by averaging the values from three wells containing PBS and dye only. The background-subtracted signal was then plotted as a function of temperature. The data was fit to a curve defined as the sum of three Gaussians, and the melting temperatures were reported as the mean of the Gaussian with the lowest peak (3).

## 3 Results

### 3.1 Affinity maturation of an Aβ conformational antibody

We have previously reported the isolation of a single-chain Aβ conformational antibody (clone 97, **Supplementary Figure S1**) selected against Aβ amyloid fibrils (3). This single-chain antibody fragment (scFv), as an Fc fusion protein, displayed i) lower affinity for Aβ fibrils, ii) higher conformational specificity for Aβ fibrils relative to Aβ monomer, and iii) much lower non-specific binding to non-Aβ proteins than a clinical-stage antibody with conformational specificity (aducanumab). To improve the potential of the 97 antibody for diagnostic and therapeutic applications, we sought to affinity mature it with the goal of achieving i) higher affinity than aducanumab, ii) similar or higher conformational specificity for Aβ fibrils relative to the 97 antibody, and iii) similar or even lower levels of non-specific binding relative to the 97 antibody. At the same time, we aimed to develop a methodology for antibody affinity maturation against insoluble antigens, such as amyloid fibrils, that is compatible with MACS and can be used by others to accomplish similar affinity maturation campaigns for antibodies specific to insoluble antigens.

Therefore, we designed a multisite CDR sub-library of the 97 antibody that included mutations in a total of 10 sites, namely five sites in LCDR2 and five sites in HCDR1, as summarized in **Supplementary Figure S1**. The use of multisite libraries is important to achieve large increases in affinity in a single library sorting campaign. The selection of these ten sites is based on a lack of mutations introduced previously into these CDRs in the initial discovery of this antibody. Given the important contributions of tyrosine to antibody affinity and specificity (25–27), and aspartic acid to low non-specific binding (3, 28–30), we mutated CDR sites in which tyrosine or aspartic acid occurred at >2% in human antibody repertoires and the wild-type residue occurred <65% (see Methods for more detail) (16).

This library was encoded as a single-chain library on yeast as Aga2-scFv fusion proteins, and sorted for binding to Aβ fibrils via MACS in rounds 1-3 (**Supplementary Figure S2**). This led to strong enrichment of the library, as a small fraction of the library was recovered after round 1 (<0.1%), but this increased after rounds 2 (~0.4%) and 3 (~2%). Next, to eliminate antibody variants that lack conformational specificity, the library was sorted by fluorescence-activated cell sorting (FACS) for a lack of binding to Aβ monomer. This revealed a mixture of antibodies with high and low levels of binding to Aβ monomer, and the bottom 10% of antibody-expressing cells with the weakest binding to Aβ monomer were collected. Finally, the antibody library was further enriched against Aβ fibrils using MACS for four additional rounds (5–8) with increased stringent washing in rounds 6, 7, and 8 to favor selection of the highest affinity variants. We increased the stringency of washing post-primary antigen incubation by incubating yeast bound to Aβ fibril-coated beads in PBSB with 0.05% Tween 20. Due to increased stringent washing, the number of cells collected dropped in rounds 6 (~0.7%) and 7 (0.2%) compared to round 5 (~2.5%). The number of cells collected in round 8 (~0.6%) was similar to round 6, suggesting that we enriched for antibodies with higher affinity.

**Figure 2:**
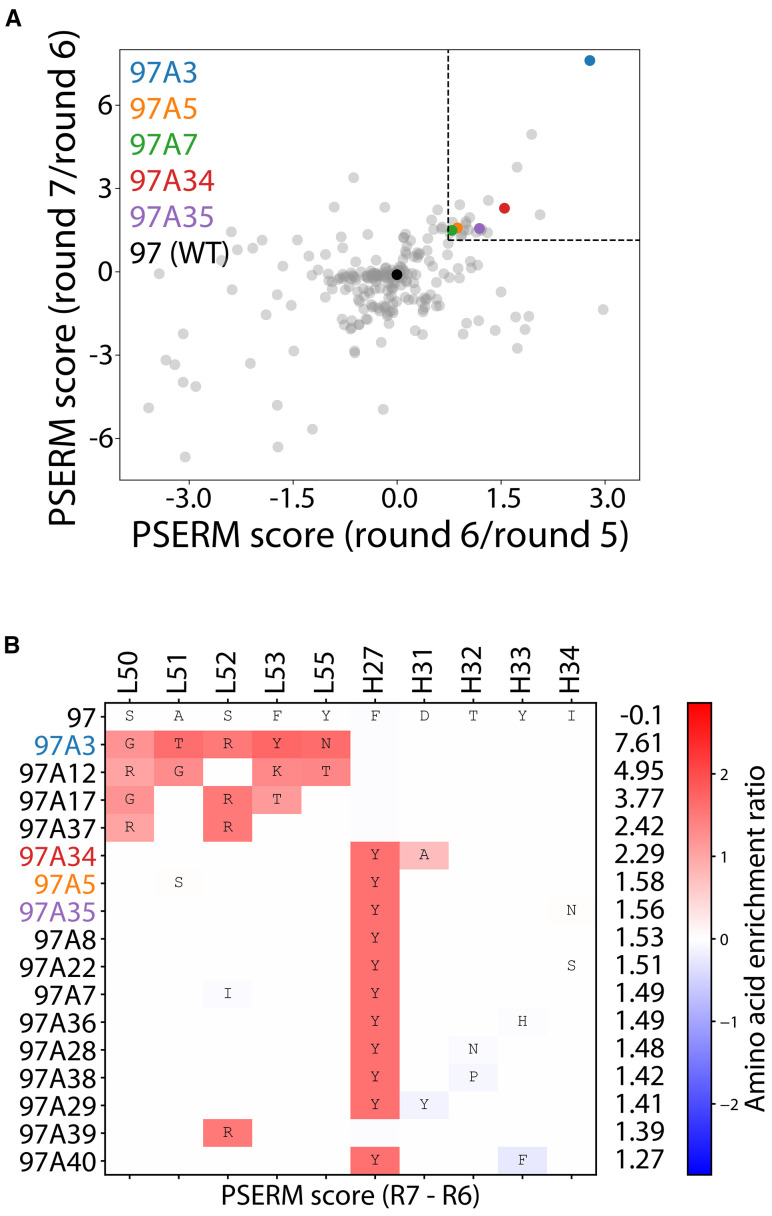
Deep sequencing analysis of enriched libraries for identifying Aβ antibodies with high affinity and conformational specificity. **(A, B)** The enriched libraries were deep sequenced after rounds 2–3 and 5-7, and the sequencing data from rounds 5-7 were used to create Position-Specific Enrichment Ratio Matrices (PSERMs). **(A)** PSERM scores were used to identify the most promising clones that displayed high values for both difference matrices. Of the 244 mutants identified, which were observed in rounds 2, 3, 5, and 6, the clones with the top 40 scores from both matrices (marked with the dotted box) were chosen for further evaluation. **(B)** For the selected clones, large positive values (dark red) signified strong enrichment of a given residue at a specific CDR site, while large negative values signified a strong depletion of a given residue at a specific CDR site. In **(A, B)**, the PSERMs were used to score individual antibody clones by calculating the difference in Position-Specific Scoring Matrix (PSSM) score at each of the ten mutated sites and then summing the scores over the ten sites.

We next deep sequenced the enriched libraries from the output of the positive selection rounds 2–3 and 5-7. The deep sequencing data were used to generate a position-specific scoring matrix (PSSM) for rounds 5-7. The data revealed that the library was primarily the wild-type sequence (~90% for each round sequenced), resulting in the highest PSSM scores for wild-type residues (**Supplementary Figure S3**). To reduce the effect of the wild-type residues, we used PSSMs from consecutive rounds of sorting to compute the difference in PSSMs, which are referred to as ΔPSSMs, that resulted in two position-specific enrichment ratio matrices (PSERMs) (21), including a PSERM for round 7 relative to round 6 and for round 6 relative to round 5 (**Supplementary Figure S4**). We then scored each clone with the two PSERMs and identified clones that scored highly for each PSERM (**Figure 2A**). To select clones for further analysis, we identified clones that ranked in the top 40 scores for both PSERMs, which resulted in a total of 16 clones (**Figure 2B**). We also repeated the PSERM analysis by first eliminating the wild-type sequence, which revealed that the PSERM scores with and without wild-type were highly correlated and resulted in the identification of similar best-scoring clones (**Supplementary Figure S5**).

We next sought to evaluate 5 of the 16 identified antibodies as soluble IgGs. Therefore, we cloned the wild type (clone 97) and mutant IgGs into heavy and light chain plasmids with human kappa IgG1 frameworks. Two control clinical-stage Aβ antibodies (aducanumab and crenezumab) were also cloned with the same human kappa IgG1 frameworks. All IgGs were expressed in HEK 293-6E cells, purified by Protein A affinity chromatography, and evaluated by analytical SEC and SDS-PAGE. The antibodies displayed high purity and expected size on SDS-PAGE gels (**Supplementary Figure S6**).

**Figure 3:**
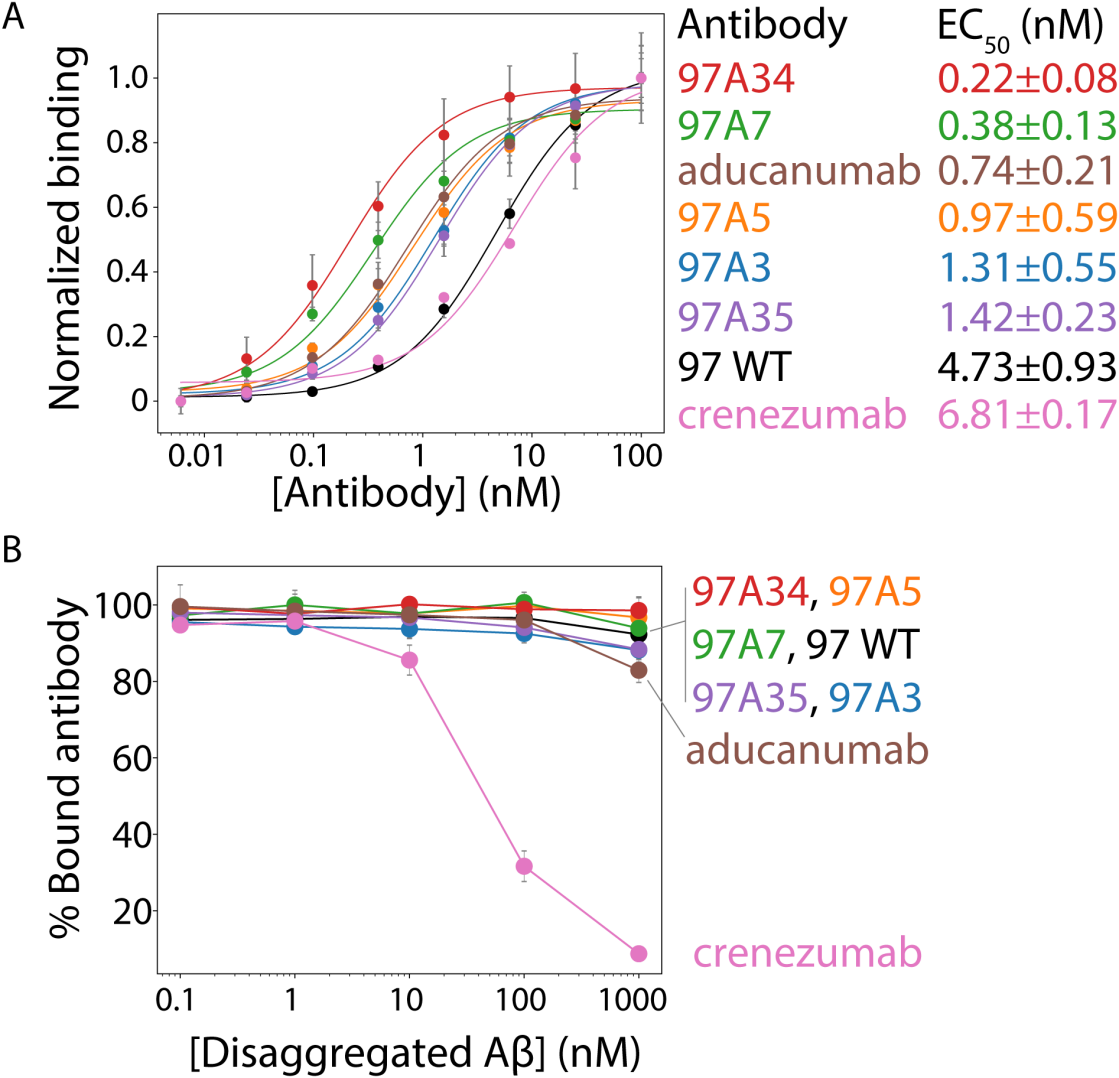
Evaluation of the relative affinities and conformational specificities of Aβ conformational antibodies. **(A)** The relative affinities of the selected antibodies were evaluated using flow cytometry analysis of soluble IgGs binding to Aβ fibrils immobilized on micron-sized magnetic beads (Dynabeads). Soluble IgGs (0.1–100 nM) were incubated with beads coated with Aβ fibrils (PBS with 0.1% BSA and 1% milk) and detected using anti-Fc Alexa Fluor 647 antibodies. **(B)** The conformational specificities of the Aβ IgGs were evaluated using flow cytometry and fibril-coated magnetic beads. The IgGs (10 nM) were first incubated with disaggregated Aβ (0.1–1000 nM), followed by incubation with Aβ fibril-coated beads (PBS with 0.1% BSA and 1% milk). Finally, the bound antibody was detected via flow cytometry using anti-Fc Alexa Fluor 647. Two clinical-stage antibodies were included as controls, namely aducanumab and crenezumab. These IgGs contain the variable regions of the clinical-stage antibodies and the constant regions from a common IgG1 framework. Therefore, the reported clinical-stage antibodies contain sequence differences relative to the actual antibody drugs. In **(A)**, the EC_50_ values are significantly lower (*p*-value <0.05) for 97A34, 97A7, 97A5, and 97A3 than for WT (97). In **(B)**, the % bound antibody at 1000 nM is statistically higher (*p*-value <0.05) for clones 97A34 and 97A7 than for aducanumab. In **(A, B)**, the binding curves are averages from two independent experiments, and the error bars are standard deviations.

Next, we tested the relative affinity of the antibodies for Aβ fibrils (**Figure 3A**, **Supplementary Figure S7**). All of the antibody variants show statistically significant improvements in affinities (EC_50_ of ~0.2–1 nM) compared to the wild type (97 WT, EC_50_ of ~5 nM) and higher affinities than the clinical antibody crenezumab (EC_50_ ~ 7 nM). Moreover, two antibodies, 97A34 and 97A7, showed higher affinity (EC_50_ of ~0.2-0.4 nM) than aducanumab (EC_50_ of ~0.7 nM).

We evaluated different approaches for identifying antibodies with improved affinity (**Supplementary Figure S8**). We compared PSERM scoring to traditional, frequency-based scoring metrics, including clonal frequency (rounds 6 and 7; R6 and R7), global enrichment ratio (R6/R2 and R7/R2), and local enrichment ratio (R6/R5 and R7/R6). Of the five variants we studied, PSERM scoring was the only method to select all five clones across the terminal two rounds of sorting. Frequency scoring failed to identify clones 97A5, 97A7, and 97A34 in the top performers. Global enrichment ratio failed to identify clones 97A5 and 97A34 across all rounds. Local enrichment ratio failed to identify clone 97A3 in both rounds. Overall, PSERM analysis demonstrated superior performance in consistently selecting improved antibody variants compared to traditional frequency-based metrics.

### 3.2 Affinity-matured Aβ antibodies have superior combinations of affinity and specificity relative to clinical-stage Alzheimer’s antibodies

We next evaluated the conformational specificity of our antibodies using a flow cytometry, bead-based assay (**Figure 3B**) (1). Trade-offs between antibody affinity and specificities are common (31–33), and we wanted to evaluate whether the increase in affinity was at the cost of specificity. Therefore, we first pre-incubated disaggregated Aβ monomer (0.1–1000 nM) with antibodies (10 nM) followed by incubation with Aβ fibrils. We then evaluated the amount of antibody bound to Aβ fibrils. Our engineered antibodies demonstrated high levels of conformational specificity, with >90% antibody bound to fibril beads even at the highest disaggregated Aβ concentration (1000 nM). This binding was higher compared to control antibodies, namely crenezumab (~10%) and aducanumab (~85%). The high conformational specificity of our antibodies is notable because disaggregated Aβ (1000 nM) was present in a 100-fold (molar excess) over the antibody (10 nM) at the highest concentration of the former species.

Next, we evaluated our antibodies using an immunodot blot assay for binding to brain homogenates from transgenic 5x FAD mice and wild-type mice (**Supplementary Figure S9**). All of our antibodies (affinity-matured clones and wild type) showed specific binding to samples from transgenic 5x FAD mice, with little binding to samples from wild-type mice. Encouragingly, the binding signal of our antibodies to 5x FAD samples appeared similar to that for aducanumab and NAB228. Surprisingly, crenezumab showed no binding to 5x FAD samples in this assay format and concentration (50 nM IgG).

To further investigate the Aβ species recognized by our antibodies using brain samples from transgenic 5x FAD mice, we evaluated western blots using two of our highest-affinity antibodies (97A7 and 97A34; **Supplementary Figure S10**). Our antibodies bound strictly to high molecular species. This result is consistent with the binding of the wild-type antibody, 97 (3).

**Figure 4:**
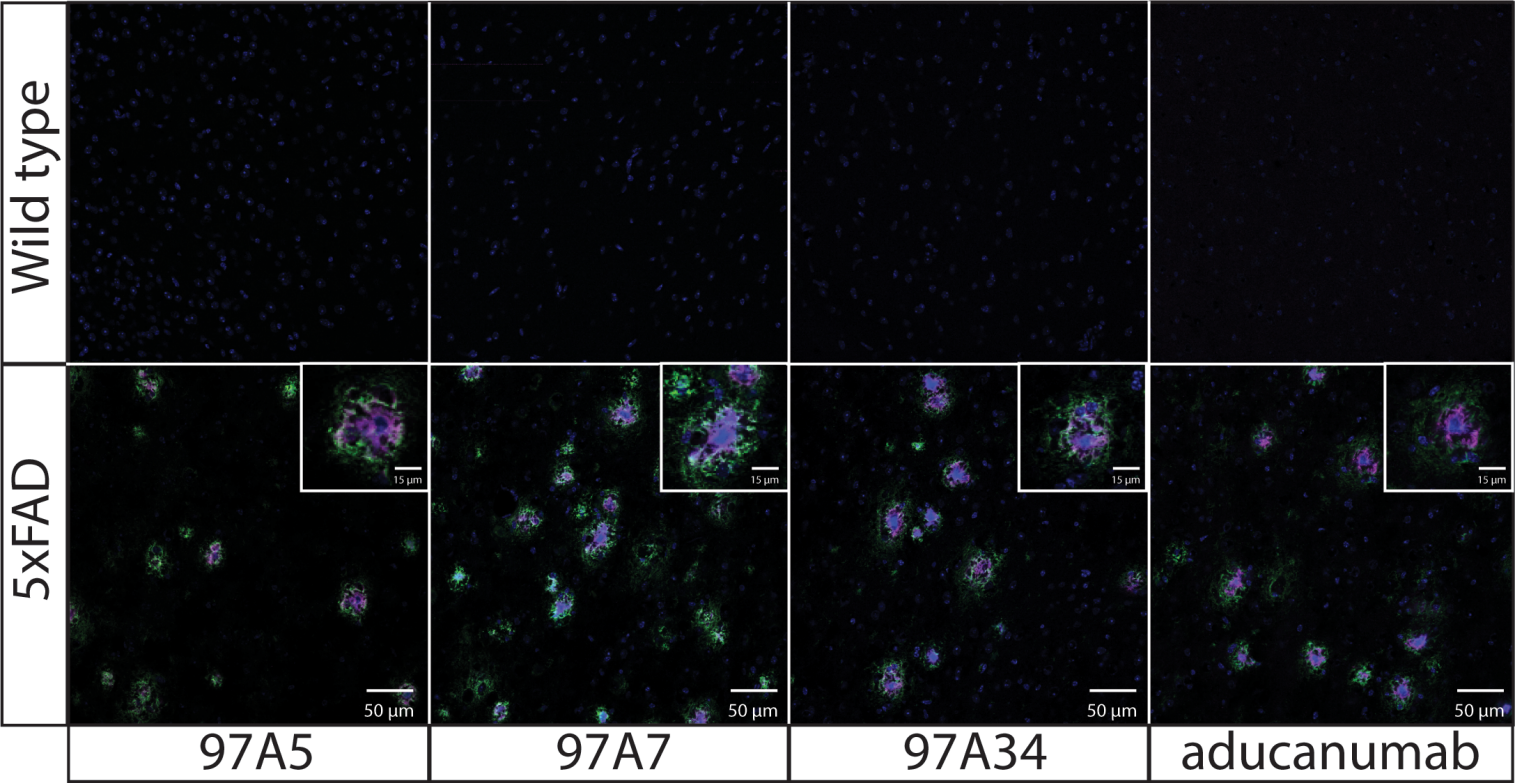
Immunofluorescence staining of transgenic 5x FAD mouse brain tissues with Aβ antibodies. Fixed brain tissues from 5x FAD transgenic and wild-type mice were stained with DAPI (blue), a panel of conformational antibodies (97A5, 97A7, 97A34, and aducanumab; purple), and a nonconformational Aβ antibody (NAB228; green). The tissue samples were incubated with 97A5, 97A7, 97A34, and aducanumab (10 nM) or with NAB228 (200x dilution) overnight at 4 °C. The conformational antibodies were detected via anti-human Fc Alexa Fluor 647, and NAB228 was detected via anti-mouse Fc Alexa Fluor 488. The images are 50 μm, and the inset images are 15 μm.

Next, we evaluated the specificity of our antibodies using immunostaining of 5xFAD transgenic mouse brain tissue (**Figure 4**). We co-stained the brain tissues from 5x FAD and wild-type mice with DAPI, conformational antibodies (97A5, 97A7, 97A34, and aducanumab), and a sequence-specific antibody (NAB228). Notably, we observed detection of Aβ aggregates for 5x FAD samples compared to wild-type samples. Interestingly, the conformational antibodies – including our antibodies and aducanumab – bound to the core of Aβ plaques (purple), while the non-conformational antibody (NAB228) bound to the periphery of the Aβ plaques (green). Similar results have been observed for other Aβ conformational antibodies that bind to the N-terminus of the Aβ peptide (34).

**Figure 5:**
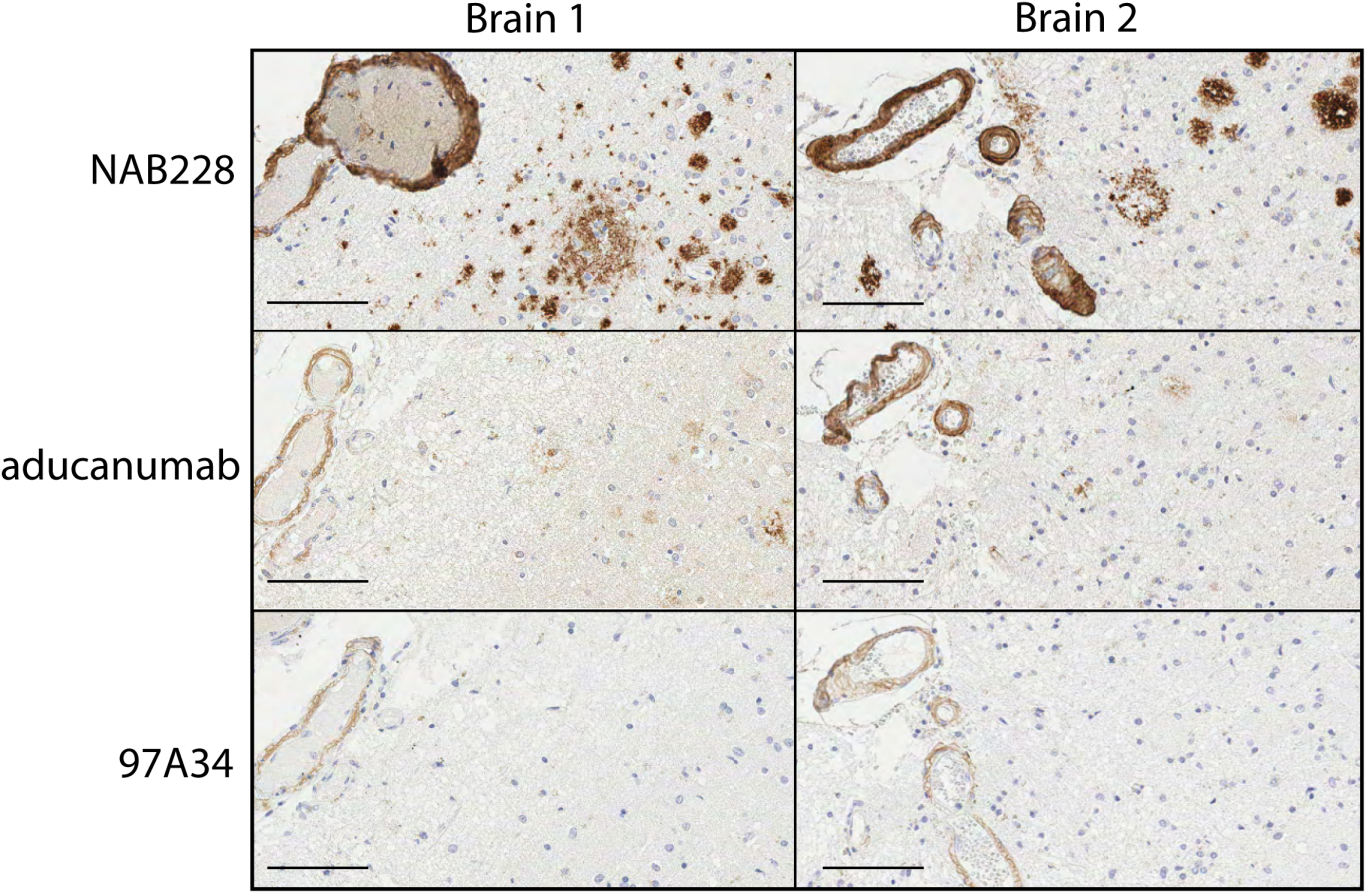
Immunohistochemical staining of human brain tissues from frontal cortex with Aβ antibodies. Staining of two different Alzheimer’s disease human brain samples, Braak 5 (left) and Braak 6 (right), was performed with (top) NAB228, (middle) aducanumab, and (bottom) 97A34. Nuclei were stained with hematoxylin. Horseradish peroxidase was used to detect the presence of antibodies and was developed using 3,3’-diaminobenzidine (DAB). Scale bars are 100 μm.

We next sought to evaluate the ability of our antibodies to recognize Aβ aggregates in human brain samples. First, we performed immunohistochemistry analysis of human brain tissues from Alzheimer’s patients with moderate cerebral amyloid angiopathy (CAA; **Figure 5**). The brains were stained with conformational (97A34 and aducanumab) and non-conformational (NAB228) antibodies. The non-conformational antibody (NAB228) showed high levels of staining throughout the tissue, including plaques and leptomeningeal vessel walls. In contrast, aducanumab stained fibrils and the walls of the leptomeningeal vessels, while 97A34 preferentially stained leptomeningeal vessel walls relative to Aβ plaques. We were able to identify staining of Aβ plaques with 97A34 in brain 1 (**Supplementary Figure S11**), although this was rare. Finally, the immunohistochemistry samples were analyzed in a blinded manner using established semi-quantitative techniques for Aβ plaque burden and CAA (35, 36), revealing that 97A34 staining corresponded to high CAA grades (scores of 2-3) and a low CERAD score (0), the latter of which is indicative of low neuritic plaque density. NAB228 showed similar CAA grades (2-3) and the highest CERAD score (2), while aducanumab displayed similar CAA grades (2-3) and an intermediate CERAD score (1).

We also evaluated the specificity of our antibodies using lysates from human Alzheimer’s disease brains (**Supplementary Figure S12**). For this assay, we generated the conformational antibodies (97A7, 97A34, and aducanumab) with mouse IgG2a Fc to avoid cross-reactivity with human antibodies in the brain samples. Encouragingly, our antibodies (97A7 and 97A34) displayed specificity for Alzheimer’s versus healthy brain lysates, and this specificity was similar to that for aducanumab, as detected via immunodot analysis.

### 3.3 Affinity-matured Aβ antibodies have drug-like biophysical properties

**Figure 6:**
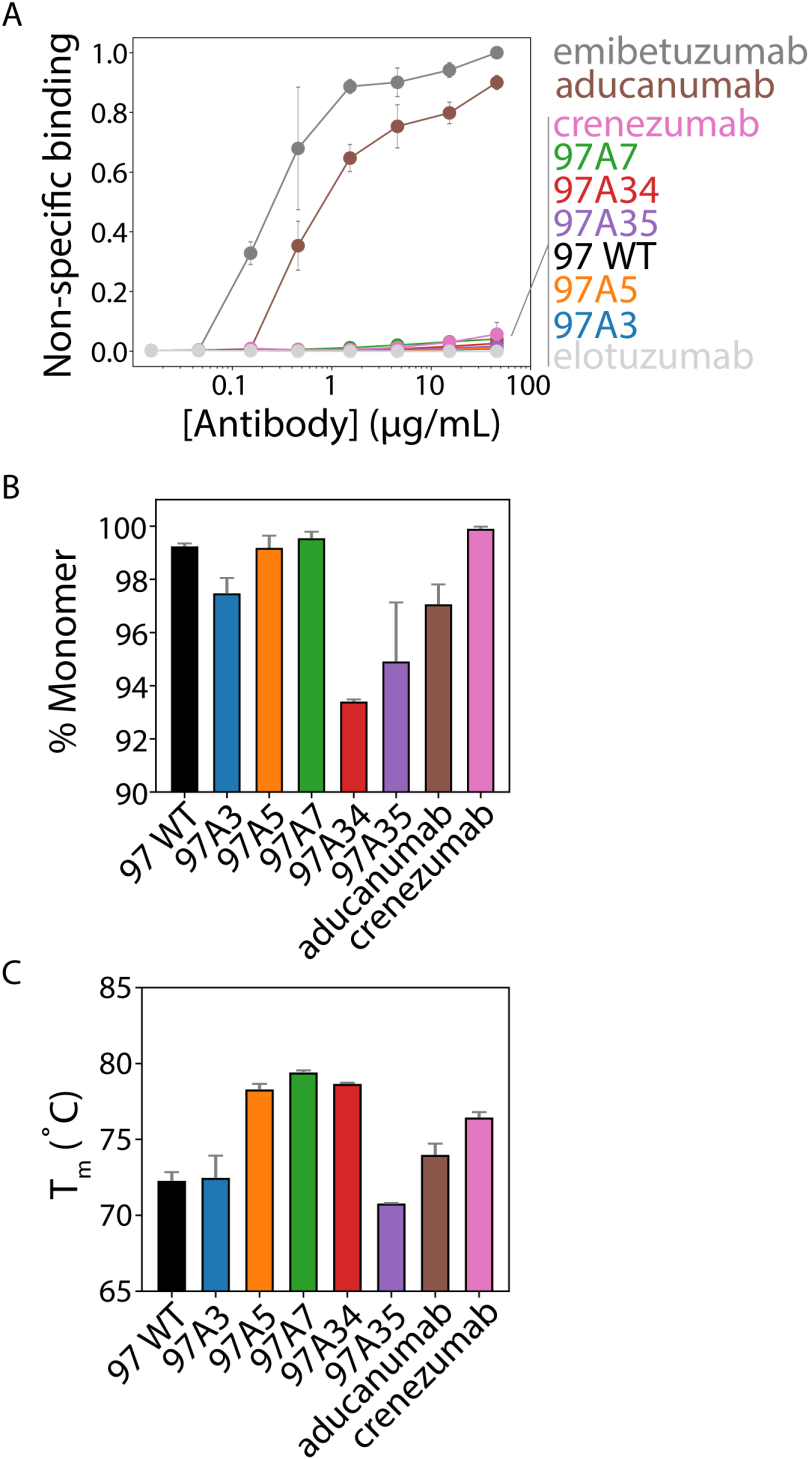
Biophysical analysis of Aβ conformational antibodies. **(A)** Nonspecific binding of Aβ antibodies to biotinylated soluble membrane proteins from CHO cells was measured via flow cytometry using IgGs immobilized on magnetic beads. Clinical-stage antibody controls were included for high (emibetuzumab) and low (elotuzumab) non-specific binding. These two control antibodies were used to normalize the levels of non-specific binding for the Aβ antibodies. **(B)** The percentage of monomeric protein (area under the curve) was evaluated by analytical size-exclusion chromatography after one-step (Protein A) purification. **(C)** Antibody melting temperatures (midpoint of first unfolding transition) evaluated using dynamic scanning fluorimetry. In **(A-C)**, the data are averages of **(A)** three, **(B)** two, and **(C)** two independent experiments, and the error bars are standard deviations.

We also evaluated our antibodies for several antibody developability properties, including their folding stability, non-specific binding, and aggregation propensity, relative to clinical-stage antibodies (**Figure 6**). First, we evaluated non-specific binding to soluble membrane proteins isolated from CHO cells (2) using a flow cytometry assay (3) (**Figure 6A**). Notably, our antibodies showed minimal non-specific binding, which was similar to that for multiple clinical-stage antibodies, including crenezumab and elotuzumab (3, 23, 37). Notably, aducanumab showed high levels of non-specific binding, which was similar to another clinical-stage antibody (emibetuzumab).

We also evaluated the purity of the antibodies after single-step purification via Protein A chromatography (**Figure 6B**). All antibodies showed relatively high levels of monomeric protein (>90%), as judged by analytical size-exclusion chromatography. Moreover, we evaluated the folding stability of our antibodies using differential scanning fluorimetry (**Figure 6C**). All antibodies demonstrated relatively high stability, with melting temperatures >70°C. Overall, these findings demonstrate that our engineered antibodies, which have higher affinity and conformational specificity than multiple Aβ clinical-stage antibodies (aducanumab and crenezumab), also have similar or better developability properties.

## 4 Discussion

We began with the goal of enhancing the affinity of our lead clone 97 while maintaining or improving conformational specificity and minimizing off-target binding. Affinity maturation for antibodies is routinely performed using FACS and has been reported against several different targets of interest (38–41). Affinity maturation against amyloid aggregates is less commonly reported (3, 4, 42) due to the complex nature of the antigen. We addressed this problem by employing MACS using micron-sized magnetic beads (i.e., Dynabeads) coated with amyloid fibrils (43). A key aspect of affinity maturation is to sequentially reduce the antigen concentration over successive rounds, thereby increasing the selection pressure to isolate high-affinity binders. The multivalent nature of amyloid aggregates hinders this process and further complicates selections for affinity. We were able to partially address this issue by reducing the concentration of aggregates immobilized on Dynabeads used for selections. Additionally, we performed multiple stringent washes in mildly harsh buffers (PBS with BSA and Tween 20) after positive selections to discourage antibodies with high off-rates, thereby attempting to select antibodies with high affinity. We substantially enhanced the affinity of our engineered clones (3-20-fold), demonstrating the utility of this approach.

Most clinical-stage antibodies are IgGs or IgG-like molecules due to their superior properties, but discovering and engineering them *in vitro* in IgG-like formats remains challenging. We have demonstrated an approach in which the lead antibody is discovered and engineered as an scFv, but reformatted and characterized in a final IgG1 format. In some cases, reformatting scFvs to IgGs can lead to a reduction in affinities (4). Notably, we did not observe this problem for our panel of antibodies; in fact, the clone 97A3 showed similar affinity and conformational specificity when evaluated as both an scFv-Fc (3) and an IgG (**Figure 3A**).

Given common trade-offs between antibody affinity and specificity (31–33), we typically observe in related discovery campaigns that clones exhibiting favorable combinations of properties, such as high affinity, high conformational specificity, and low nonspecific binding, are rare. Furthermore, Sanger sequencing of our terminal sorting round yielded mainly wild-type 97 sequences, and deep sequencing revealed that the wild-type sequence was the dominant clone, accounting for ~90-95% abundance in all sequenced rounds. Thus, the need for deep sequencing analysis and methods for selecting clones was paramount for successfully finding improved variants. Non-wild-type variants had very few sequences, which increases the uncertainty of frequency-based metrics used for antibody selection.

PSERM scoring alleviated this issue by leveraging all the sequencing data to generate sequence scores, allowing for the selection of extremely rare clones with relatively high accuracy. It was notable that even on this small scale, PSERM effectively identified valuable variants within a theoretical diversity of 10^20^, despite our limited sequencing depth of only 10^6–^10^7^ reads. This significant sampling bottleneck, combined with the dominance of a single clone that represents ~90-95% of sequences, creates substantial challenges for traditional frequency-based selection approaches. Our method demonstrates that meaningful scoring and ranking of rare variants is possible even when they represent a minute fraction of the total library. For example, our best variants (97A7 and 97A34) were present at extremely low frequency during selection, including at 0.0008-0.001% in round six and 0.0012-0.0043% in round seven. These findings highlight that our approach may enable efficient mining of the vast sequence space in antibody libraries where the most desirable clones may exist at extremely low frequencies.

It is notable that our antibodies recognize Aβ aggregates in the brains of both transgenic mice and humans with Alzheimer’s disease and moderate CAA, even though our library selections were performed using only synthetic Aβ peptide. Moreover, it is also interesting that our antibodies show specificity for staining the walls of leptomeningeal vessels instead of the plaques throughout human brain tissue from Alzheimer’s patients with moderate CAA (**Figure 5**, **Supplementary Figure S11**). This may suggest that Aβ deposits in leptomeningeal vessels – a hallmark of CAA (44, 45) – are unique from those in Aβ plaques in the brain. Indeed, the structures of Aβ40 fibrils isolated from the leptomeninges of either sporadic AD or CAA were solved using cryo-EM, revealing a structured N-terminus and a protofibril structure in which residues Asp-1 to Gly-38 form four β-strands (46). In contrast, structures of Aβ42 fibrils isolated from the brains of AD patients reveal that the N-terminal 8–10 residues are disordered (46). This difference is notable because the epitope of the parental antibody in this study (clone 97) – which recognizes both Aβ40 and Aβ42 fibrils – involves the first three residues of the Aβ N-terminus (3), and the unique properties of the N-terminus of Aβ fibrils in leptomeningeal vessels relative to the brain may explain the conformational specificity of our antibodies, although more work is needed to test this speculative observation.

The fact that our antibodies have desirable developability properties also deserves further consideration. Antibody nonspecific binding, or polyreactivity, is a major concern when developing antibodies as drugs due to the risk of fast antibody clearance (47–49). Our affinity-matured antibodies show low levels of nonspecific binding, even compared to their parental antibody (clone 97), which is notable because we were able to maintain high specificity while performing affinity maturation against a highly charged and hydrophobic antigen (Aβ fibrils). This result is also notable because we did not perform any counter selections against non-specific reagents, in contrast to other related studies (4, 24). Instead, we performed only positive selections against Aβ fibrils in a relatively complex environment (PBS with BSA and milk) to reduce nonspecific binding, as well as a negative selection against Aβ monomer. This resulted in affinity-matured antibodies with affinities and conformational specificities similar to those of aducanumab, but with significantly less nonspecific binding.

The origin of the low nonspecific binding of our affinity-matured antibodies relative to aducanumab appears to be due to large differences in their CDR compositions. It is notable that the net charge of antibody CDRs is a key determinant of nonspecific binding, as more positively charged CDRs are linked to increased nonspecific binding (28, 29, 50). Aducanumab has an unusual HCDR3 sequence (95- DRGIGARRGPYYMD-102), including three positively charged (arginine) residues, while clone 97 and the affinity-matured variants lack such positively charged residues in HCDR3 (95-DGYDGSYFVGYDYNDFYDY-102). The net charge of the six CDRs for aducanumab is 3.1 (pH 7.4), while it is -2.8 for clone 97 and -2.8 to -1.8 for the affinity-matured variants (97A3, 97A5, 97A7, 97A34, and 97A35). Moreover, it is interesting that most of our affinity-matured variants, including the ones with the highest affinities, contained a Phe-H27-Tyr mutation, highlighting the importance of tyrosine in mediating specific antibody recognition. While we did not limit amino acid diversity at each mutated site in the CDRs, these findings open the door to future studies with designed amino acid diversity to bias toward CDR amino acid compositions with optimal combinations of affinity, specificity, and other drug-like properties.

## Data Availability

Data reported in this publication is available at https://github.com/Tessier-Lab-UMich/druglike-abeta-antibodies. IHC images are available on request.

## Supplementary material

Supplementary Figure 1 Amino acid sequence of parental clone 97 and summary of antibody library design. **(A)** Variable heavy (V_H_) and variable light (V_L_) domains of parental clone 97 with CDRs marked in blue. **(B)** To affinity mature the parental antibody (97 WT), five positions in light chain CDR2 and five positions in heavy chain CDR1 were mutated using NNK codons.

Supplementary Figure 2 Sorting summary for the selection of Aβ conformational antibodies. **(A)** An antibody sub-library of clone 97 was displayed on the surface of yeast as single-chain antibody fragments (scFvs) and subjected to seven rounds of positive sorting against Aβ fibrils via magnetic-activated cell sorting (sorts 1–3 and 5-8). The percentage of cells retained after each positive selection relative to the input (10^9^ cells for round 1 and 10^7^ cells for the remaining rounds) is shown. **(B)** In sort 4, the library was sorted negatively against disaggregated Aβ (1000 nM) by FACS to eliminate antibodies with strong binding to Aβ monomer by collecting antibody-displaying cells in the R7 gate.

Supplementary Figure 3 Position-specific scoring matrices (PSSMs) for the deep sequencing data from rounds 5-7. The enriched libraries were deep sequenced after rounds 5-7, and the sequencing data were used to create position-specific scoring matrices (PSSMs). Large positive values (dark red) signify a high observed frequency of a given residue at a specific CDR site, while large negative values signify a low observed frequency of a given residue at a specific CDR site.

Supplementary Figure 4 Position-specific enrichment ratio matrices (PSERMs) for scoring clones. The deep sequencing data were used to generate PSERMs for scoring clones. Large positive values (dark red) signify high enrichment of an amino acid at a specific CDR site, while large negative values signify depletion of an amino acid at a specific CDR site.

Supplementary Figure 5 Comparison of deep sequencing analysis of enriched Aβ antibody libraries with and without the wild-type antibody sequence. **(A, B)** The enriched libraries were deep sequenced after rounds 5-7, and the sequencing data were used to create PSERMs from rounds 5–7 with and without the WT antibody sequence, given that WT was frequently observed. The PSERMs calculated from consecutive rounds were computed. **(A)** PSERM scores with and without the WT sequences were well correlated. **(B)** PSERM scores identify some of the same most promising clones that displayed high values for both matrices (97A3, 97A34, and 97A35), regardless of whether WT sequences were included, while other clones (97A5 and 97A7) were only identified when including the WT sequences. See **Figure 2** for additional details.

Supplementary Figure 6 SDS-PAGE analysis of the Aβ IgGs evaluated in this study. Purified IgGs were evaluated prior to heating and reduction (-) and after heating and reduction (+). The gels (10% Bis-Tris) were visualized using Coomassie blue staining.

Supplementary Figure 7 Statistical analysis of the EC_50_ values of the WT and affinity-matured antibodies. EC_50_ values for each antibody that are significantly lower than 97 (WT) are marked with “*”, corresponding to *p*-values <0.05.

Supplementary Figure 8 Alternative metrics for selecting Aβ conformational antibodies. **(A)** The relative affinity of each selected clone is shown and compared to WT. **(B-E)** Deep sequencing metrics are shown for each variant and WT for both round 6 (R6, darker bars) and round 7 (R7, lighter bars). The scoring metrics evaluated were **(B)** PSERM scoring, **(C)** clonal frequency, **(D)** global enrichment ratio (ER), defined as the log2 transform of the frequency of a clone in round 6 or 7 divided by its frequency in round 2, and **(E)** local ER, defined as the log2 transform of the frequency of a clone in round 6 or 7 divided by its frequency in the previous round. In **(B-E)**, the filled bars show clones that have scores in the top 40 clones for each metric in each round. In other words, the bars for enrichment ratios for clone 97A3 are filled because the enrichment ratios for this variant were in the top 40 values in both rounds 6 and 7. The error bars are propagated errors, and the *p*-values (* is <0.05) are calculated relative to WT (clone 97).

Supplementary Figure 9 Immunodot blot analysis of Aβ antibody detection of brain homogenates from transgenic 5x FAD and wild-type mice. Brain homogenates from transgenic 5x FAD and wild-type (control) mice were first immobilized on nitrocellulose membrane, followed by incubation with IgGs at 50 nM (TBS with 0.1% Tween 20 and 1% milk) overnight at 4 °C. The signals were detected using chemiluminescence. Ponceau-stained blot was used as a loading control (LC). Two clinical-stage antibodies, aducanumab (Adu) and crenezumab (Cre), were included as controls. A sequence-specific antibody (NAB228), which recognizes both soluble and aggregated Aβ, was also included as a control. The experiments were performed three times, and a representative example is shown.

Supplementary Figure 10 Western blot analysis of Aβ antibodies using brain samples from transgenic 5x FAD mice. Brain homogenates from transgenic 5x FAD and wild-type (control) mice were first run on SDS-PAGE, followed by transfer onto nitrocellulose membranes. Next, the membranes were incubated with IgGs at 100 nM (97A7 and 97A34) or 1000x dilution (NAB228) in TBS with 0.1% Tween 20 and 1% milk overnight at 4 °C. The signals were detected using chemiluminescence. Ponceau-stained blot was used as a loading control (LC). A sequence-specific antibody (NAB228), which recognizes both soluble and aggregated Aβ, was also included as a control. The experiments were performed three times, and a representative example is shown.

Supplementary Figure 11 Immunohistochemical staining of human brain tissues with Aβ antibodies. Additional regions of two brain sections from Braak stage 5 (left/middle) and stage 6 (right) brain sections stained with (top) NAB228, (middle) aducanumab, and (bottom) 97A34. Hematoxylin nuclear counterstain; horseradish peroxidase detection with DAB development.

Supplementary Figure 12 Immunodot blot analysis of Aβ antibody detection of homogenates from Alzheimer’s disease human brains. Homogenates from Alzheimer’s disease (AD) and healthy (control) human brains were first immobilized on nitrocellulose membranes and then incubated with Aβ antibodies [97A7 and 97A34 at 100 nM, aducanumab (adu) at 10 nM, and NAB228 at 1000x dilution] in PBST with 1% milk overnight at 4 °C. The signals were detected using chemiluminescence. The experiments were performed three times, and a representative example is shown. Ponceau-stained blot was used as a loading control (LC).
