# Supplementary material for: Directed evolution of drug-like Aβ conformation-specific antibodies
Source: Front Immunol. 2025 Oct 13;16:1655893. doi: 10.3389/fimmu.2025.1655893 (PMC12554751; doi:10.3389/fimmu.2025.1655893)
Supplement: Supplementary Figure 1 — Amino acid sequence of parental clone 97 and summary of antibody library design. (A) Variable heavy (VH) and variable light (VL) domains of parental clone 97 with CDRs marked in blue. (B) To affinity mature the parental antibody (97 WT), five positions in light chain CDR2 and five positions in heavy chain CDR1 were mutated using NNK codons. [file DataSheet1.pdf]

A

Clone 97

V_H EVQLVESGGGLVQPGGSLRLSCAASGFNIKDTYIHWVRQAPGKGLEWVARIYPASGATRYADSV
KGRFTISADTSKNTAYLQMNSLRAEDTAVYYCARDGYDGSYFVGYDYNDFYDYWGQGTLVTVSS

V_L DIQMTQSPSSLSASVGDRVTITCRASQNVAYAVTWYQQKPGKAPKLLIYSASFLYSGVPSRFSGS
RSGTDFTLTISSLQPEDFATYYCQQHSTYPPTFGQGTKVEIK

B

	LCDR2							HCDR1									
Position	50	51	52	53	54	55	56	26	27	28	29	30	31	32	33	34	35
Wild-type	**S**	**A**	**S**	**F**	**L**	**Y**	**S**	**G**	**F**	**N**	**I**	**K**	**D**	**T**	**Y**	**I**	**H**
Mutated	**X**	**X**	**X**	**X**	-	**X**	-	-	**X**	-	-	-	**X**	**X**	**X**	**X**	-
WT Residue (%)	5	42	65	<1	24	<1	74	88	48	2	10	<1	10	2	17	19	25
Tyrosine (%)	3	<1	2	<1	<1	<1	<1	<1	18	<1	1	3	2	62	17	6	6
Aspartic acid (%)	20	11	4	5	<1	2	1	1	7	<1	<1	3	10	1	4	<1	<1

Figure S1. Amino acid sequences of parental clone 97 and summary of antibody library design. (**A**) Variable heavy (V_H) and variable light (V_L) domains of parental clone 97 with CDRs marked in blue. (**B**) To affinity mature the parental antibody (97 WT), five positions (X) in light chain CDR2 and five positions in heavy chain CDR1 were mutated using NNK codons.

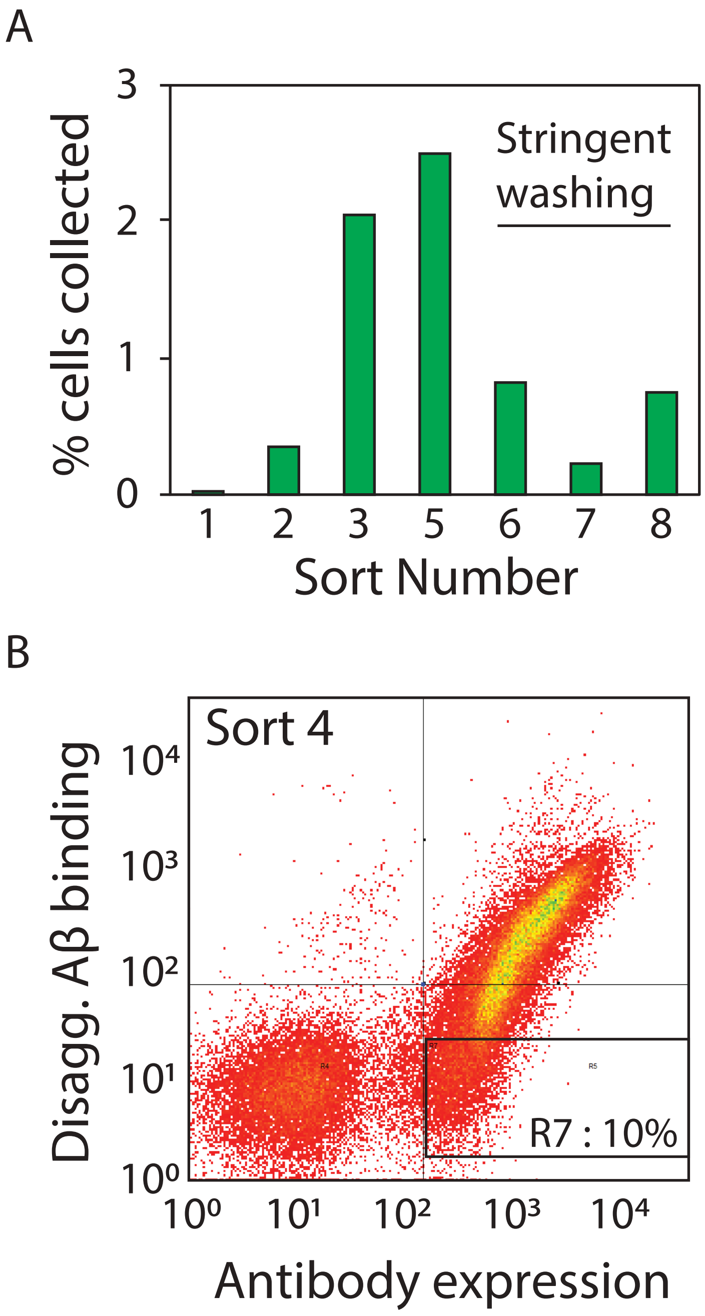

Figure S2. Sorting summary for the selection of Aβ conformational antibodies. (A) An antibody sub-library was designed with degenerate NNK codons at five sites in light chain CDR2 and five sites in heavy chain CDR1. The library was displayed on the surface of yeast as single-chain antibody fragments (scFvs) and subjected to seven rounds of positive sorting against Aβ fibrils via magnetic-activated cell sorting (sorts 1-3 and 5-8). The percentage of cells retained after each positive selection relative to the input (10^9 cells for round 1 and 10^7 cells for remaining rounds) are shown. (B) In sort 4, the library was sorted negatively against disaggregated Aβ (1000 nM) by fluorescence-activated cell sorting (FACS) to eliminate antibodies with strong binding to Aβ monomer by collecting antibody-displaying cells in the R7 gate.

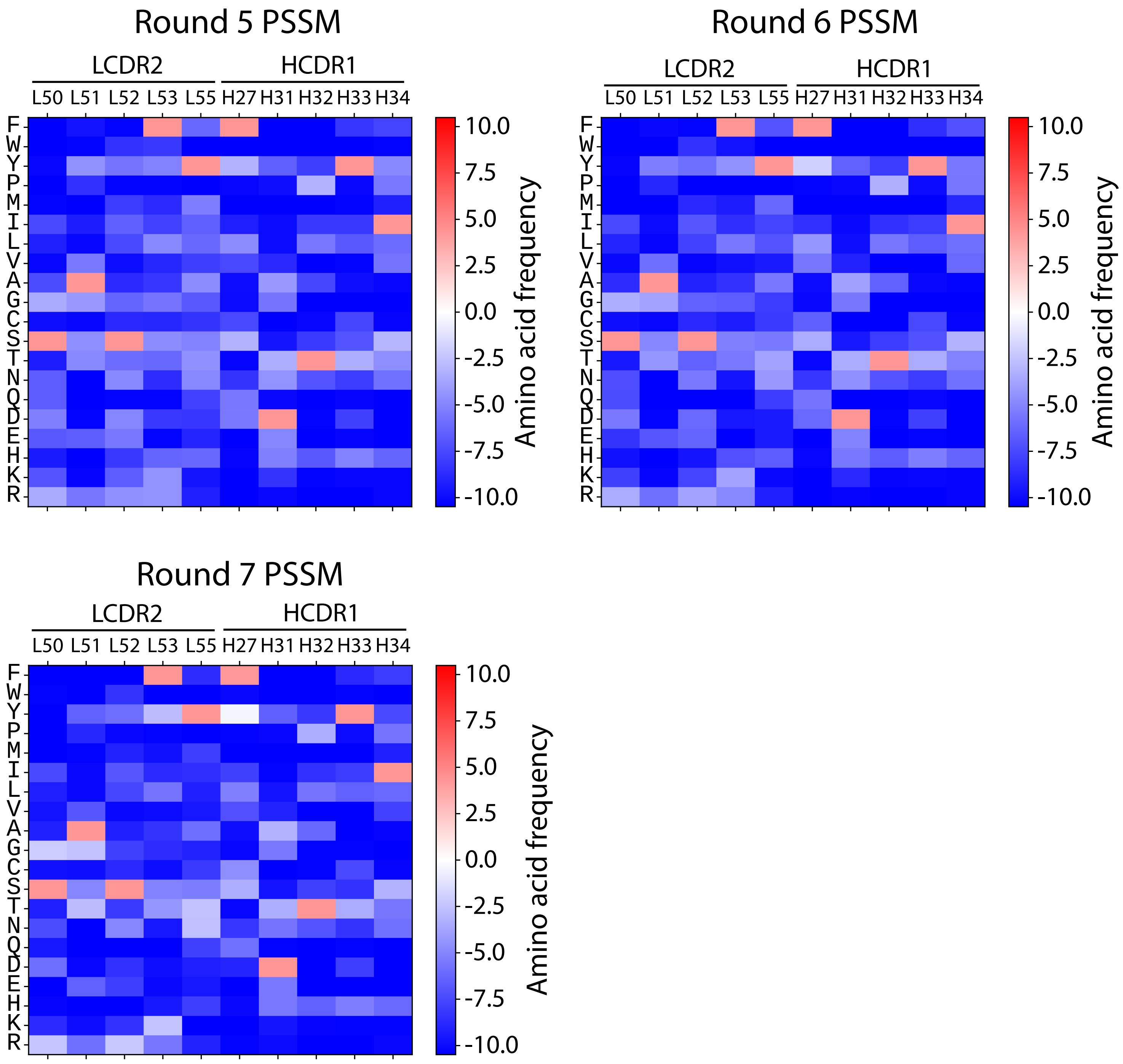

Figure S3. Position-specific scoring matrices (PSSMs) for the deep sequencing data from rounds 5-7. The enriched libraries were deep sequenced after 5-7, and the sequencing data was used to create position-specific scoring matrices (PSSMs). Large positive values (dark red) signified strong enrichment of a given residue at a specific CDR site, while large negative values signified a strong depletion of a given residue at a specific CDR site.

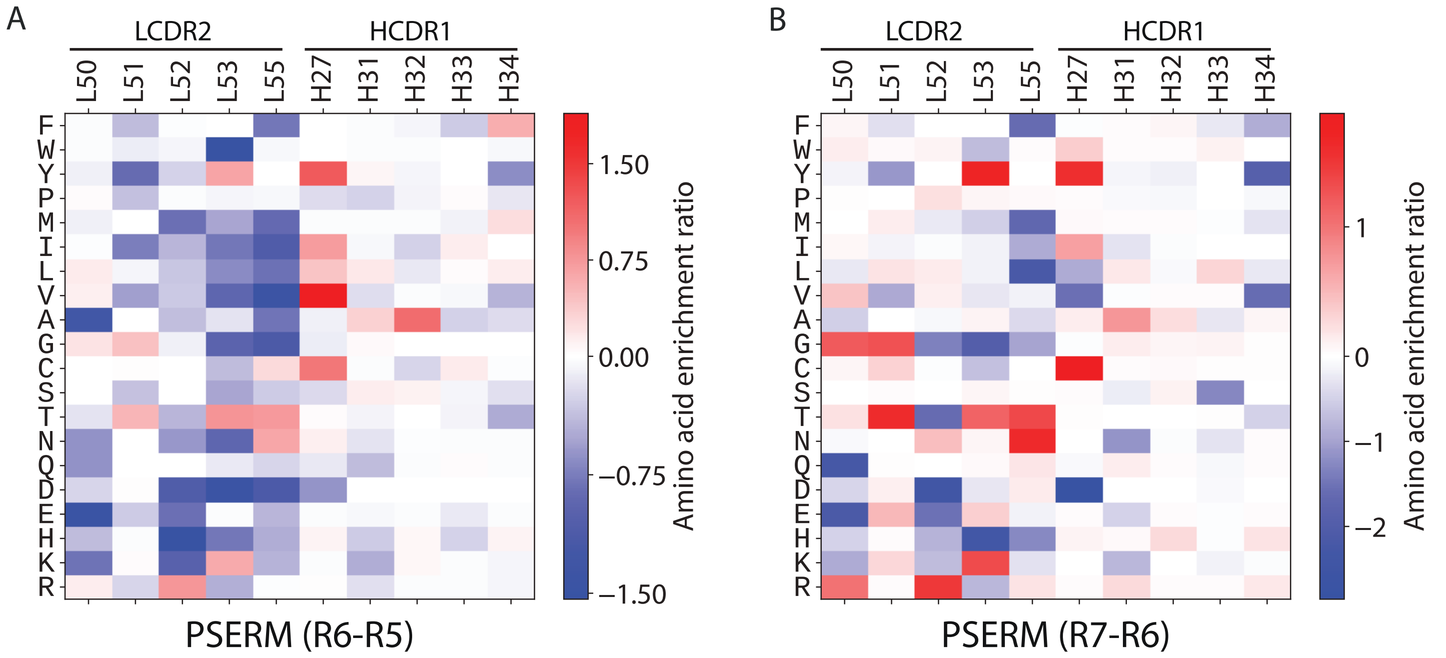

Figure S4. Position-specific enrichment ratio matrices (PSERMs) for scoring clones. The deep sequencing data was used to generate Position Specific Enrichment Ratio Matrices (PSERM) for scoring clones. Large positive values (dark red) signify high enrichment of an amino acid at a specific CDR site, while large negative values signify depletion of an amino acid at a specific CDR site.

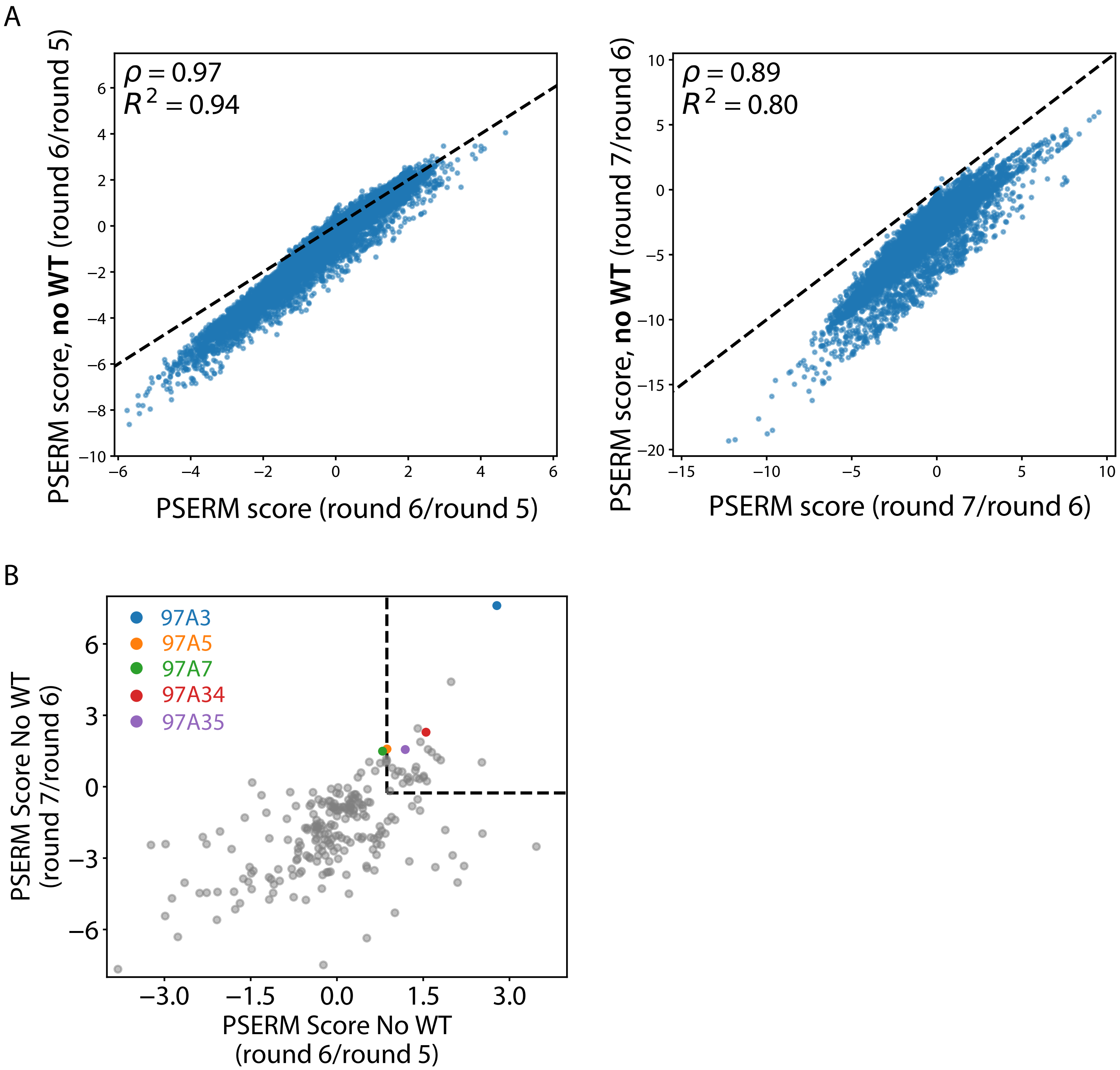

Figure S5. Comparison of deep sequencing analysis of enriched Aβ antibody libraries with and without the wild-type antibody sequence. (**A-B**) The enriched libraries were deep sequenced after rounds 5-7, and the sequencing data were used to create PSERMs from rounds 5-7 with and without the WT antibody sequence, given that WT was frequently observed. The PSERMs calculated from consecutive rounds were computed. (**A**) PSERM scores with and without the WT sequences were well correlated. (**B**) PSERM scores identify some of the same most promising clones that displayed high values for both matrices (97A3, 97A34, and 97A35), regardless of whether WT sequences were included, while other clones (97A5 and 97A7) were only identified when including the WT sequences. See Figure 2 for additional details.

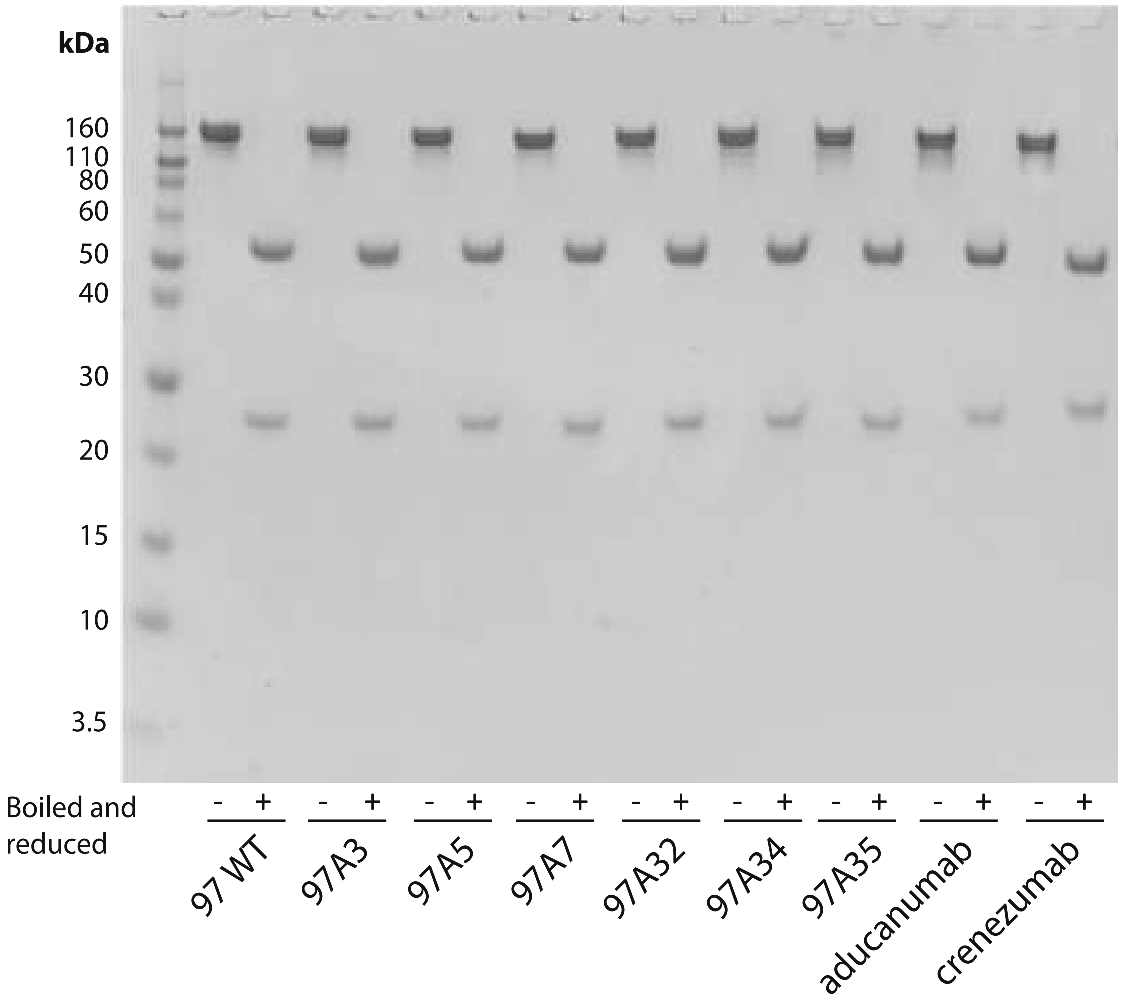

Figure S6. SDS-PAGE analysis of the Aβ IgGs evaluated in this study. Purified IgGs were evaluated prior to heating and reduction (-) and after heating and reduction (+). The gels (10% Bis-Tris) were visualized using Coomassie blue staining.

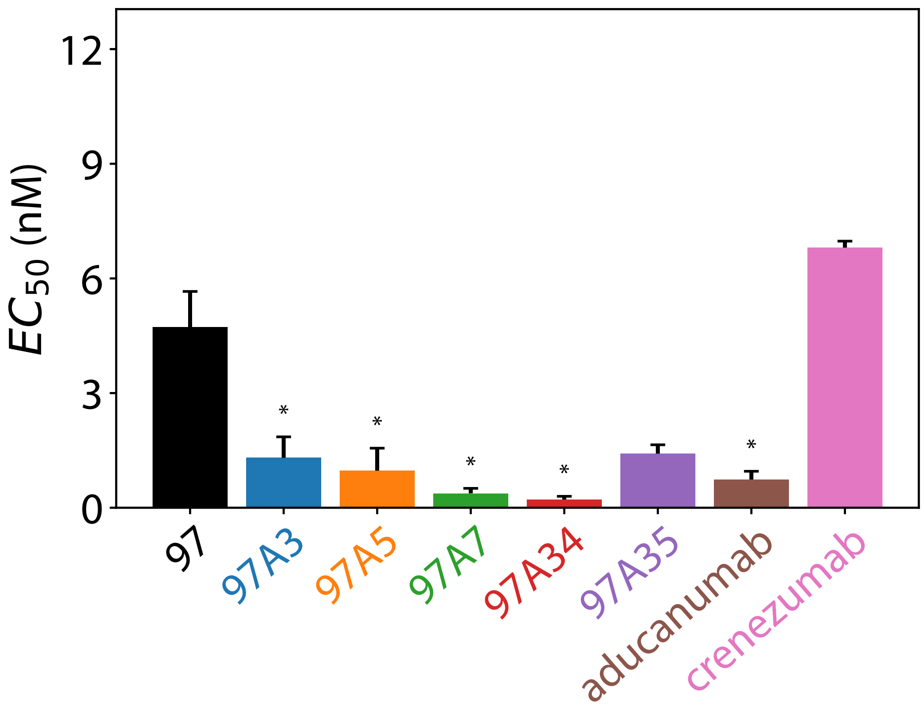

Figure S7. Statistical analysis of the EC_{50} values of the WT and affinity-matured antibodies. EC_{50} values for each antibody that are significantly lower than 97 (WT) are marked with "*", corresponding to *p*-values <0.05.

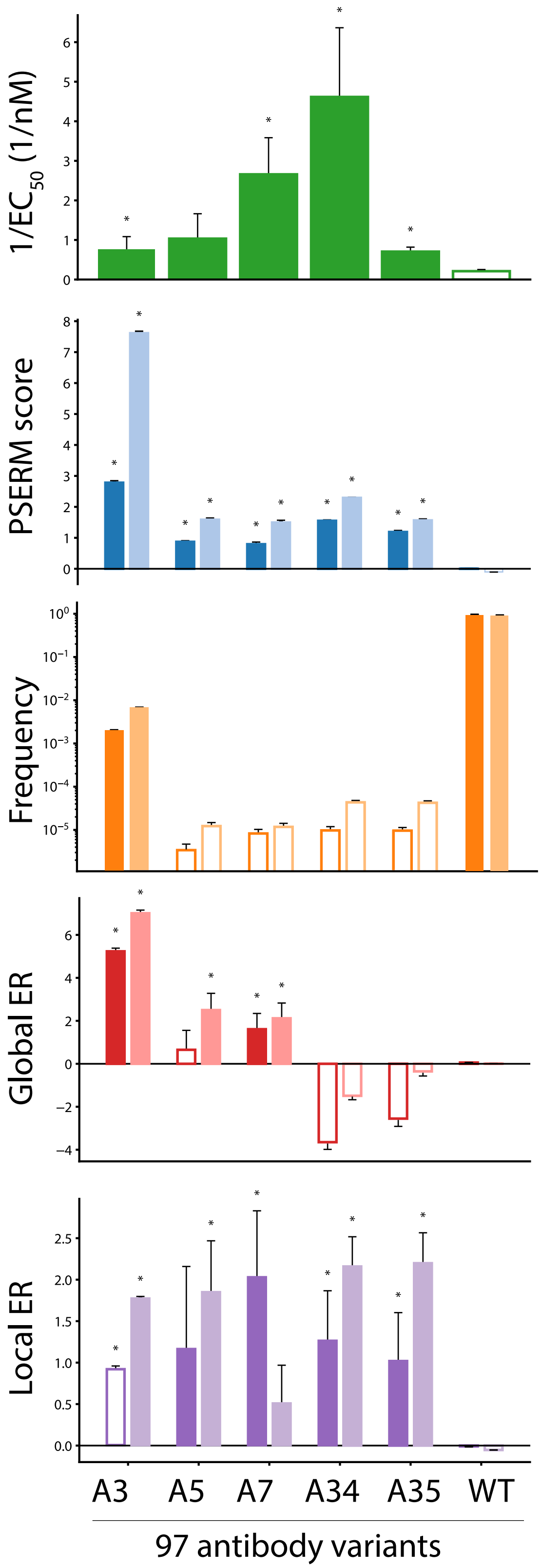

Figure S8. Alternative metrics for selecting Aβ conformational antibodies. (**A**) The relative affinity of each selected clone is shown and compared to WT. (**B-E**) Deep sequencing metrics are shown for each variant and WT for both round 6 (R6, darker bars) and round 7 (R7, lighter bars). The scoring metrics evaluated were (**B**) PSERM scoring, (**C**) clonal frequency, (**D**) global enrichment ratio (ER), defined as the log2 transform of the frequency of a clone in round 6 or 7 divided by its frequency in round 2, and (**E**) local ER, defined as the log2 transform of the frequency of a clone in a round 6 or 7 divided by its frequency in the previous round. In (**B-E**), the filled bars show clones that have scores in the top 40 clones for each metric in each round. In other words, the bars for enrichment ratios for clone 97A3 are filled because the enrichment ratios for this variant were in the top 40 values in both rounds 6 and 7. The error bars are standard deviations, and the p-values (* is <0.05) are calculated relative to WT (clone 97).

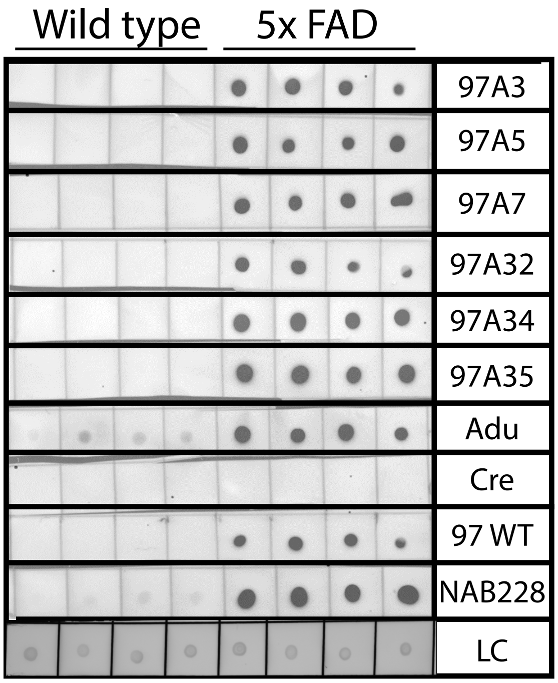

Figure S9. Immunodot blot analysis of Aβ antibody detection of brain homogenates from transgenic 5x FAD and wild-type mice. Brain homogenates from transgenic 5x FAD and wild type (control) mice were first immobilized on nitrocellulose membrane followed by incubation with IgGs at 50 nM (TBS with 0.1% Tween 20 and 1% milk) overnight at 4 °C. The signals were detected using chemiluminescence. Ponceau stained blot is used as a loading control (LC). Two clinical-stage antibodies, aducanumab (Adu) and crenezumab (Cre), were included as controls. A sequence-specific antibody (NAB228), which recognizes both soluble and aggregated Aβ, was also included as a control. The experiments were performed three times and a representative example is shown.

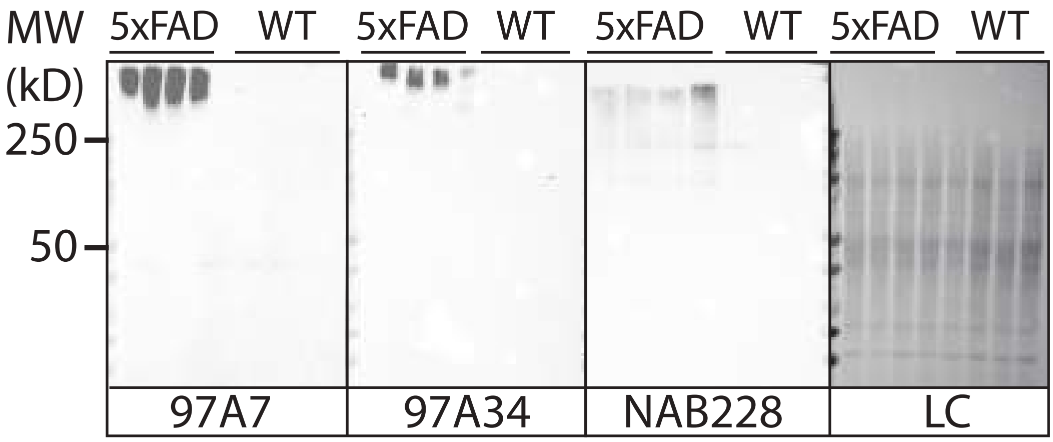

Figure S10. Western blot analysis of Aβ antibodies with transgenic 5x FAD mice. Brain homogenates from transgenic 5x FAD and wild type (control) mice were first run on SDS-PAGE followed by transfer onto nitrocellulose membranes. Next, the membranes were incubated with IgGs at 100 nM (97A7 and 97A34) or 1000x dilution (NAB228) in TBS with 0.1% Tween 20 and 1% milk overnight at 4 ˚C. The signals were detected using chemiluminescence. Ponceau stained blot is used as a loading control (LC). A sequence-specific antibody (NAB228), which recognizes both soluble and aggregated Aβ, was also included as a control. The experiments were performed three times and a representative example is shown.

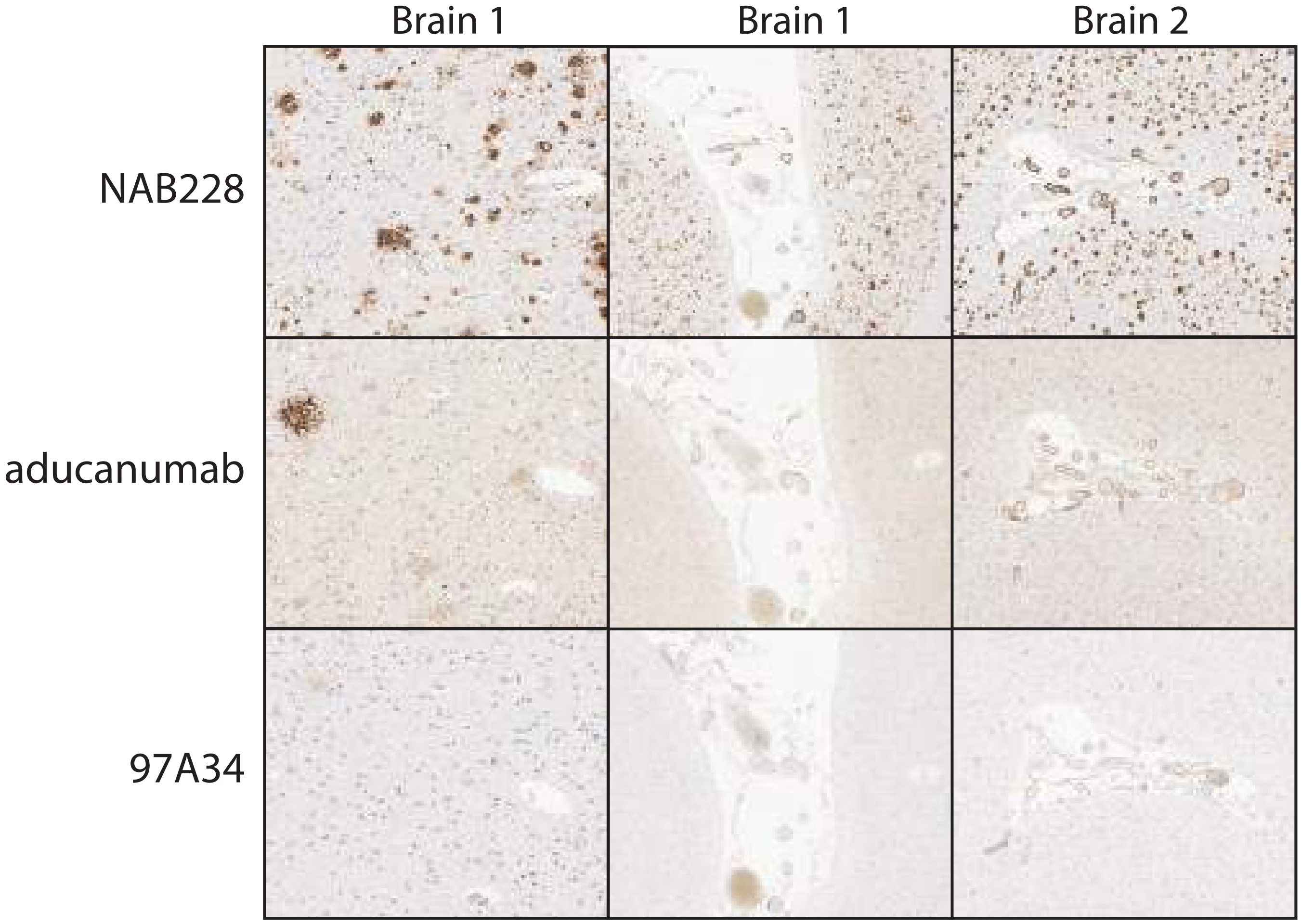

Figure S11. Immunohistochemical staining of human brain tissues from frontal cortex with Aβ antibodies. Additional regions of two brain sections for Braak stage 5 (left/middle, brain 1) and stage 6 (right, brain 2) stained with (top) NAB228, (middle) aducanumab, and (bottom) 97A34. Hematoxylin nuclear counterstain; horseradish peroxidase detection with DAB development.

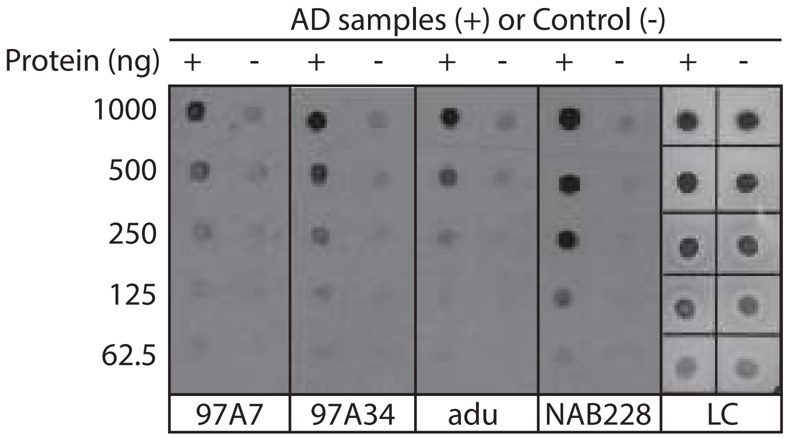

Figure S12. Immunodot blots analysis of Aβ antibody detection of homogenates from Alzheimer's disease human brains. Homogenates from Alzheimer's disease (AD) and healthy (control) human brains were first immobilized on nitrocellulose membranes and then incubated with Aβ antibodies (97A7 and 97A34 at 100 nM, aducanumab (adu) at 10 nM, and NAB228 at 1000x dilution) in PBST with 1% milk overnight at 4 ˚C. The signals were detected using chemiluminescence. The experiments were performed three times and a representative example is shown. Ponceau stained blot is used as a loading control (LC).
